# Dry-Jet Wet Spinning of Thermally Stable Lignin-Textile Grade Polyacrylonitrile Fibers Regenerated from Chloride-Based Ionic Liquids Compounds

**DOI:** 10.3390/ma13173687

**Published:** 2020-08-20

**Authors:** Muhannad Al Aiti, Amit Das, Mikko Kanerva, Maija Järventausta, Petri Johansson, Christina Scheffler, Michael Göbel, Dieter Jehnichen, Harald Brünig, Lucas Wulff, Susanne Boye, Kerstin Arnhold, Jurkka Kuusipalo, Gert Heinrich

**Affiliations:** 1 Institute of Materials Science, Technische Universität Dresden, D-01062 Dresden, Germany; lucaswulff@gmx.de; 2 Leibniz-Institut für Polymerforschung Dresden e. V., D-01069 Dresden, Germany; das@ipfdd.de (A.D.); scheffler@ipfdd.de (C.S.); goebel@ipfdd.de (M.G.); djeh@ipfdd.de (D.J.); bruenig@ipfdd.de (H.B.); boye@ipfdd.de (S.B.); Arnholdk@ipfdd.de (K.A.); 3 Polymer Science Engineering, Materials Science and Environmental Engineering, Faculty of Engineering and Natural Sciences, Tampere University, FI-33014 Tampere, Finland; mikko.kanerva@tuni.fi; 4 Paper Converting and Packaging Technology, Materials Science and Environmental Engineering, Faculty of Engineering and Natural Sciences, Tampere University, FI-33014 Tampere, Finland; maija.jarventausta@tuni.fi (M.J.); petri.johansson@tuni.fi (P.J.); jurkka.kuusipalo@tuni.fi (J.K.); 5 Institut für Textilmaschinen und Textile Hochleistungsfaserstofftechnik, Technische Universität Dresden, D-01062 Dresden, Germany

**Keywords:** lignin, dry-jet wet spinning, precursor fibers, stabilization kinetics, fiber structure formation, entropy elastic shrinkage, semi-crystalline structure

## Abstract

In this paper, we report on the use of amorphous lignin, a waste by-product of the paper industry, for the production of high performance carbon fibers (CF) as precursor with improved thermal stability and thermo-mechanical properties. The precursor was prepared by blending of lignin with polyacrylonitrile (PAN), which was previously dissolved in an ionic liquid. The fibers thus produced offered very high thermal stability as compared with the fiber consisting of pure PAN. The molecular compatibility, miscibility, and thermal stability of the system were studied by means of shear rheological measurements. The achieved mechanical properties were found to be related to the temperature-dependent relaxation time (consistence parameter) of the spinning dope and the diffusion kinetics of the ionic liquids from the fibers into the coagulation bath. Furthermore, thermogravimetric analysis (TGA), differential scanning calorimetry (DSC), and dynamic mechanical tests (DMA) were utilized to understand in-depth the thermal and the stabilization kinetics of the developed fibers and the impact of lignin on the stabilization process of the fibers. Low molecular weight lignin increased the thermally induced physical shrinkage, suggesting disturbing effects on the semi-crystalline domains of the PAN matrix, and suppressed the chemically induced shrinkage of the fibers. The knowledge gained throughout the present paper allows summarizing a novel avenue to develop lignin-based CF designed with adjusted thermal stability.

## 1. Introduction

Carbon fiber (CF) reinforced thermoplastics and thermosets combine high stiffness and high strengths with low density, enabling them as light-weight construction materials for space and aviation sectors, wind energy, and automotive industry.

The high tensile strength and high tensile modulus of CF are owing to the unique preferably along with the fiber axis oriented turbostratic or graphite-like carbonaceous crystal structure. However, the characteristic and unavoidable porosity, as well as the pore dimensions and misorientation of the pores with respect to the fiber main axis, govern, to a wide extent, the mechanical performance of the fibers. In order to achieve certain tensile strengths and tensile moduli of CF, defined structural parameters should be maintained. Some of these parameters are as follows:

- crystal thickness L_c_;
- crystal width parallel L_a//_ and perpendicular L_a┴_ to the fiber axis in meridional and equatorial modes;
- interlayer spacing of the basal planes d_002_;
- relative orientation degree and Herman’s orientation factors of the crystals with respect to the fiber main axis;
- porosity;
- true pore length, overall tilt angle (misorientation) of the pores with respect to the fiber main axis;
- specific surface of the pores;
- intersection length and radius of gyration;
- orientation degree of the pores alongside the fiber main axis.

The structural characteristics can be adjusted utilizing over 200 manufacturing parameters of the precursor system used and the manufacturing process. In one of our last papers, we reported in detail about the structure–property relation of CF and the impact of the manufacturing process on the resulting properties [1].

The global demand of CF increases yearly at a rate of 10–13% (2010–2017) [2,3,4,5]. Co-polymers of polyacrylonitrile constitute as precursors ca. 90% of the global CFCF market. Ten percent of the CF is made from mesophase pitch and isotropic pitch. One of the critical drawbacks of CF was high costs, making the breakthrough of this material in the low-market segments, that is, the automotive industry, more difficult. The precursor costs of polyacrylonitrile account for slightly more than 50% of the costs of PAN-based CF [5]. CF with reduced costs should also maintain a certain level of mechanical properties, that is, tensile strength >1.7 GPa, tensile modulus >210 GPa, as well as elongation at break >1% [6].

From the economic viewpoint, different research methodologies were recently reported [6]. For instance, utilization of heavy tow textile grade acrylic fibers prepared by pseudo-melt spinning technology and stabilization via advanced oxidative stabilization technique, that is, the microwave assisted plasma was reported [7].

Another important aspect of CF production is the low carbon yield (up to 50%) of the polyacrylonitrile. This means that the production capacity of a PAN-based CF plant is one half of that of PAN plant.

Lignin is a by-product of the pulping process and a low-cost material. The high abundance, one-third of the dry weight of the lignocellulosic biomass, and the aromatic character of this material are of a paramount importance for further economic utilization for high value-added products, such as CF precursors. Caused by the pulping process, Lignin showed consistency problems, making it unsuitable for fiber formation, that is, very low molecular weight values and the high polydispersity lead to brittleness of the fiber structure, and the high ash content leads to defects and flaws in the fine fiber structure.

Utilizing lignin as a precursor material for high value-added products like CF is not a new research topic. Different research routes were pursued to formulate lignin-based precursor fibers and, subsequently, lignin-based CF with high mechanical properties. To date, lignin-based CF show low mechanical performance, that is, low tensile strength < 1 GPa, low tensile modulus <150 GPa, and low elongation at break <1% [6]. Additionally, the X-ray porosity of lignin-based CF is high and exceeds 30–38% depending on the thermal conversion [8]. The low molecular weight decreases the probability of conjunction and joining zones between the carbonaceous crystals in the subsequent lignin based CF. The high ash content increases the possibility of the development of flaw defects in the fine structure of CF, and hence deteriorates the mechanical performance of the fibers. The high content of hydroxyl groups hinders the fusibility of lignin and the concentration and constitution of methoxy groups increase the glass transition region of the lignin [9,10]. This hinders in turn the processability of the material utilizing eco-friendly processes like melt spinning. In addition, the high content of hydroxyl groups and the low molecular weight (as a molecular origin) increase the agglomeration tendency of the lignin fractions even in strong solvents, that is, in the water-based binary solvent system with N, N-dimethylformamide (DMF), dimethyl sulfoxide (DMSO), or dimethylacetamide (DMAc). This leads in the wet-spinning process with polyacrylonitrile to leaching phenomena in the coagulation bath [11].

Additionally, the high hydroxyl group content of lignin increases the hydrogen bonding and hinders the fusibility of the polymers. This is the main reason why lignin cannot be melt-processed without any chemical modifications. The melt-spinning and compatibilization of lignin with different polyolefin polymers and liquid crystal polymers as a supporting matrix require a chemical derivatization, that is, alkylation or acetylation [12,13,14].

Most of the research works regarding lignin-based CF mainly considered the utilization of softwood Kraft lignin types for the following shortly summarized reasons.

Softwood Kraft lignin can be technically extracted from the black liquors with a relatively low (chemically bounded) sulfur content (1–3 wt. %), for instance, utilizing the Lignoboost^®^-Process [15], compared with the lignin extracted from the sulphite-process, for example, magnesium or sodium-based sulphite process.

Organosolv hardwood lignin is very pure (in terms of ash content) and almost sulfur free owing to the organosolv extraction process; however, the high methoxy group content was found to reduce the thermal stability of the lignin compared with the softwood lignin [16,17].

Softwood lignin is highly susceptible to any thermal processing, which initiates radical polymerization and increases the molecular weight owing to the cross-linking. This mechanism shortens the stabilization time required during the oxidative stabilization of softwood lignin-based precursor fibers [18]. Different studies report the compounding of softwood and hard wood lignin to increase the thermal stability of the mixture and enhance the thermal oxidative stabilization [19,20].

To date, lignin-based CF, regardless of the PAN/lignin-based CF, show low mechanical properties, and the achieved properties are inferior to the set targets [6].

The absence of the semi-crystalline structure of lignin prevents the orientation of the molecules alongside the fiber axis during the high shear stress extrusion through fine spinning nozzles.

Recently, Clauss et al. reported the possibility of increasing the molecular weight of lignin utilizing the reactive compounding. It is assumed that, the higher the molecular weight, the better the orientation of lignin molecules alongside the fiber axis would be. Residual chars up to 49% @ 800 °C under nitrogen purge were achieved [13].

A crucial drawback is the extremely low thermal stability of lignin [21,22] and the undefined thermal decomposition owing to the lack of semi-crystalline sequences or domains. This becomes visible in terms of higher porosity of the residual chars at elevated temperatures (above 2000 °C) and can be evaluated utilizing the µCT-measurements. Comparatively, Steudle et al. reported in their work [8] the increasing X-ray determined porosity of the lignin-based CF at high treatment temperatures. The measured porosity values reached 30–38% at a heat treatment temperature of 2000 °C. This is owing to the fact that lignin in general consists of non-repetitive or random chemical bonds, which was found to enhance the release of volatile products in the early pyrolysis region between 200 and 600 °C and to produce weakly cross-linked clusters, which are furthermore thermally unstable [21]. On the basis of our recent work, it is well known that the origin of the pores in CF is mainly caused by the amorphous regions in the precursor fibers [1,6]. The randomly constituted amorphous lignin oligomers inevitably lead to an undefined pore structure in the subsequent CF.

Recently, different research works aimed to develop continuous precursor fibers based on different trinary systems, for example, lignin/pure cellulose/ionic liquids or lignin/Kraft pulp/ionic liquids, utilizing the dry-jet wet spinning technology [23,24,25,26]. The utilization of cellulose aimed to over-compensate the low molecular weight of lignin. Strong and homogenous precursor fibers with increased thermal stability and higher carbon yield for the systems (lignin/cellulose) could be achieved. Nevertheless, not enough structural data of the fiber fine structure were reported. Moreover, the impact of lignin on the fine structure and the thermo-mechanical behavior of the fibers were not discussed in depth.

Byrne et al. [27] studied the stabilization mechanisms of cellulose/lignin fibers regenerated from the ionic liquid: 1,5-diazabicyclo(4.3.0)non-5-ene-1-ium acetate (DBNH^+^) (OAc^−^) and the impact of lignin on the stabilization using FT-IR techniques. Unfortunately, no structural data regarding the impact of lignin on the thermo-mechanical behavior of the fibers, that is, thermally induced entropic shrinkage, oxidative shrinkage, and the influence of lignin on the fine structure of the fibers, were reported.

In order to gain CF based on lignin/PAN in combination, main attention should be paid to the adjustment of the thermal stability of the starting material, as well as the ordering and orientation of the lignin oligomers alongside the fiber axis. This fundamental research methodology will be the scope of the present research paper. Additionally, the present paper focuses on the impact of ionic liquids, softwood lignin oligomers, and the processing parameters on the thermal, thermo-mechanical, and mechanical properties of the precursor fibers. Furthermore, the impact of the processing parameters and rheological data will be correlated to the fine structure of the fibers, that is, crystal sizes, interlayer spacing, relative orientation degree, and Herman’s orientation factor. The impact of lignin on the stabilization kinetics and the kinetics of the thermo-mechanical behavior of the fibers will also be discussed in depth throughout the present paper.

## 2. Materials and Methods

### 2.1. Materials

Pure ionic liquid (IL) 1-ethyl-3-methylimidazolium chloride (EMIM^+^) (Cl^−^) (Assay (NMR) >98%) was purchased from io.li.tec Ionic Liquids Technologies GmbH, D-74076 Heilbronn, Germany. The contents of the cation 1-ethyl-3-methylimidazolium and the chloride anions were 99.9% for each. The water content was found to be less than 1 wt. %.

Textile grade homo-polyacrylonitrile (HPAN) with acrylonitrile content greater than 99.5 wt. % and molecular weight of approximately 200,000 g·mol^−1^ was supplied by DOLAN GmbH, D-93309 Kelheim, Germany. Softwood BioChoice^TM^ Kraft Lignin (SBKL) was obtained from UPM Biochemicals, FI-00101 Helsinki, Finland. Softwood Kraft lignin was selected, as Softwood Kraft lignin is industrially available as lignin-type and, upon the thermal conversion process, shows lower void formation tendency, as the content of the methoxy groups is lower than the Hardwood organosolv lignin. SBKL was dried at 80 °C under vacuum for 24 h prior to further characterization, purification, and further processing. The molecular characteristics of the utilized lignin are summarized in the supplementary data.

*Ash removal from SBKL*: For CF precursors [6], lignin should be extensively purified to remove any contaminating alkali metals or free sulfur. This is of importance to avoid any possible flaws generated in the fibers by the ash, as reported by Baker et al. [28], Klett et al. [29], and Ishikawa et al. [30].

The impurities in lignin including acid soluble carbohydrate substitutes and ash have been extracted via the dialysis process [31]. Around 200 g of dried SBKL powder was dispersed under vigorous stirring at 1200 rpm in a two-liter solution of hydrochloric acid with the molarity of 2.5 M at 60 °C for one hour. The pH was controlled between 1 and 3. After washing, the SBKL was precipitated and separated from the acidic solution via a common filtration set-up using a ceramic Brüchner funnel and flask connected to a vacuum pump. Ash-free filter papers (Grade 390, Pore retention 3–5 µm) were supplied by Sartorius Stedim Biotech GmbH, D-37,079 Göttingen, Germany.

The as-separated SBKL cake was washed under rigorous stirring with four liters of hot (T = 60 °C) deionized water. After successive washing, the cake was washed off with deionized water and dried at 70 °C under vacuum for 24 h.

After drying, the remained ash content of lignin was investigated according to the norm DIN EN ISO 3451-1:2008. The ash content of the neat SBKL was 1.29 wt. % and, after the acidic washing, the ash content was reduced to 0.15 wt. %.

### 2.2. Methods

#### 2.2.1. Micro-Compounder

In order to evaluate the miscibility of the trinary system textile grade polyacrylonitrile, Biochoice^TM^ Softwood Lignin and ionic liquids the DSM Xplore^®^ micro compounder, Netherland, was utilized. The DSM micro compounder was equipped with conveying fully intermeshing twin screws and a chamber volume of 15 cm^3^, and was used to develop different compounds of lignin, textile grade PAN, and ionic liquids.

#### 2.2.2. Shear Rheological Measurements

The shear rheological measurements of the developed trinary system dopes (PAN/Softwood BioChoice^TM^/Ionic Liquids) and the binary system dopes (PAN/Ionic Liquids) were investigated utilizing the ARES G2 strain-controlled oscillatory rheometer (TA Instruments, 159 Lukens Drive, New Castle, UK) to describe their viscoelastic pseudo-flow behavior and their thermal stability. The strain resolution was 0.04 µrad and the minimum angular displacement oscillation was 1 µrad. The samples were heated in a forced convection furnace using nitrogen as a heating medium. Cone-plate geometry made from stainless steel with the diameter of 25 mm and cone angle of 0.0997 rad was used. The gap offset was 0.0559 mm, giving a sample volume of 0.4091 cm^3^.

##### Oscillatory Strain Sweep Test

To identify the spinning dopes for being linear or non-linear viscoelastic, the strain dependency of both the storage moduli (G′) and loss moduli (G″) was evaluated at a constant frequency and two different temperatures (85 °C and 105 °C). Additionally, the yield stresses of the spinning dopes were estimated for the studied dopes under the same conditions.

##### Oscillatory Frequency Sweep Test

To ensure the comparability of the data of different spinning dopes, the deformation amplitude of 6% in the linear viscoelastic range was selected for all dopes from the strain sweep tests. At this strain amplitude, frequency sweep measurements at five different temperatures with temperature increments of 10 K in the temperature range 65 °C to 105 °C were performed within a modest frequency range from 100 Hz to 0.1 Hz and a resolution of nine points per decade.

The master curves of the complex viscosities as a function to the angular frequency were displayed for five different reference temperatures.

Assuming the applicability [32] of the Cox–Merz empirical relationship [33] to the binary or trinary polymer system of the spinning dopes at low frequencies, the plot of the steady state shear viscosity η versus the shear rate $\dot{\gamma }$ was assumed to correspond to the magnitude of the complex viscosity $\left|\eta^{*}\right|$ versus the angular frequency ω:

(1) $$\left|\eta^{*}\left(\omega \right)\right|\to \eta \left(\dot{\gamma }\right)$$

From the plot of the steady state shear viscosity η versus shear rate $\dot{\gamma }$, the Carreau–Yasuda fit model was used to describe the pseudo-flow behavior and to calculate the zero shear rate viscosity as well as the consistency (relaxation time) of the spinning dope at the related five reference temperatures.

(2) $$\frac{\eta \;-\;\eta_{\infty }}{\eta_{0}\;-\;\eta_{\infty }}={\left[1+{\left(k\dot{\gamma }\right)}^{a}\right]}^{\frac{n\;-\;1}{a}}$$

where $\eta_{0}$ is the zero shear rate viscosity, $\eta_{\infty }$ is the infinite shear rate viscosity, k is the consistency parameter (characteristic relaxation time), $\dot{\gamma }$ is the shear rate, n is the power low index, and a is a parameter describing the flow transition between the Newtonian plateau and the power law region.

##### Time–Temperature–Superposition

The miscibility of the polymer blends can be evaluated via dynamic-mechanical measurements and, under certain conditions, applying the time–temperature–superposition (TTS) [34,35,36,37,38,39,40].

The TTS was evaluated from the logarithmic value of the horizontal shift factors αT of the storage modulus at 85 °C versus the term 1/T. If there was no break in the TTS plot, the system was considered miscible and the activation energy of the flow was calculated from the slope of the linear regression of the TTS plot using the Arrhenius model.

(3) $$\ln\left(a_{T}\right)=\frac{E_{AF}}{R}\left(\frac{1}{T}\;-\;\frac{1}{T_{0}}\right)$$

where $E_{AF}$ is the flow activation energy, R is the ideal gas constant (8.31448 J·K^−1^·mol^−1^), and T_0_ is the reference temperature.

#### 2.2.3. Dry-Jet Wet Spinning

Selected spinning dopes were compounded on a self-constructed vertical kneader under nitrogen purge. Prior to the spinning trails, the dopes were filtered and degassed overnight. Dry-Jet Wet spinning trails were performed with a modular laboratory scale Dry-Jet Wet spinning system at Tampere University, Finland. A titanium spinneret nozzle made from with a 300 µm cylindrical nozzle with the aspect ratio L/D of 2 and an internal opening cone angle of 60° was used. The degassed and filtered spinning dope was permanently forwarded from the sealed tank to the Zeith^®^ gear pump by nitrogen gas with the pressure of 4 bar at 85 °C. The gear pump achieved a minimum rotational speed of 0.5 rpm. This in turn allows (depending on the spinning dope viscosity) a minimum throughput of 0.08–0.09 g·min^−1^. The Dry-Jet Wet Spinning machine allowed to increase speed of the first take-up godet between 2.5 and 50 m·min^−1^. The maximum possible overall in-line stretching of the attenuated fibers was 10 times.

The spinning set-up (spinning dope tank, spinning gear, spinning tubes, and spinning nozzle) was permanently heated by a silicon heating tape system, type KM-HT-203-SO, SAF Wärmetechnik GmbH, D-69509 Mörlenbach). The temperature of the spinning set-up and spinning nozzle was permanently controlled and adjusted to 85 °C for all spinning trails. The coagulation (phase-separation process) of the attenuated filaments was performed in a deionized water bath with an initial specific conductivity of 0.5 µS·cm^−1^ at 25 °C. All spinning trials were performed at a constant air gap of 5 cm between the spinning nozzle and the surface of the water bath and a constant length of the coagulation line in the water bath of 80 cm.

The temperature of the coagulation bath was controlled via an ethylene glycol cooling unit and high surface area stainless heat exchanging unit.

The take-up zone consisted of three glass godets with corrugated surfaces. Between the first and second godets, a heated water bath (60 °C) with deionized water was used to stretch the fibers in-line. All fibers in this work were collected on the second godets under permanent washing with deionized water (23 °C).

#### 2.2.4. Single Fiber Tensile Test

Single fiber tensile test was conducted to evaluate the mechanical properties of the fibers, that is, tenacity, Young’s modulus, the elongation at break, and work to rupture, using a FAVIGRAPH from Textechno H. Stein GmbH (Mönchengladbach, Germany). The Young’s modulus was determined by the slope of the linear regression of the tenacity–strain curves at terminal values of the strain (0.05–0.5%). The measuring force cell was 100 N, the clamping length was 30 mm, and the constant strain rate was selected to be 1.39%·s^−1^ (25 mm·min^−1^) at a relative humidity of 65% and ambient temperature (23 °C). For each developed fiber type, 20 sample measurements were performed.

The FAVIGRAPH was also equipped with a vibration cell to determine the linear density (fineness) of each single fiber prior to the tensile test using the vibration method according to ASTM D 1577.

#### 2.2.5. Wide Angle X-Ray Scattering (WAXS)

The supramolecular structure of the developed as-spun fibers and the off-line hot-stretched fibers was studied by the wide angle X-ray scattering (WAXS) using the X-ray diffractometer D8 DISCOVER (Bruker AXS GmbH, Karlsruhe, Germany) with pine-hole collimation system with 0.5 mm diameter and a Cu Kα monochromatized radiation source (λ = 0.15418 nm). Large MICROGAPTM Detector (VÅNTEC-500) with a 10,000 mm^2^ gap-free active area was used. The fibers were mounted on a self-constructed Euler Cradle system in order to achieve very wide diffraction angles and to avoid any shadow effects of the clamping system during the measurements.

The crystal size of the develop fibers was calculated, depending on the orientation status of the fibers, from the meridional and equatorial reflection at 2θ~17° using the Scherrer equation:

(4) $$L_{hkl}=\frac{K\cdot \lambda }{\beta_{corr}\cdot \cos\left(\theta \right)}$$

where $L_{hkl}$ is the crystal size related to the (*hkl*) lattice plane and K is a dimensionless shape factor. For polymeric materials, it has a typical value of (0.87–1.00). In this study, K was 0.9; λ is the X-ray wavelength and is equal to 0.15418 nm; $\beta_{corr}$ is the corrected line broadening at half the maximum intensity (FWHM). $\beta_{corr}$ can be calculated from the following equation:

(5) $$\beta_{corr}=\sqrt{\beta^{2}\;-\;\beta_{MB}{}^{2}\;}$$

where β is the apparent FWHM of the (*hkl*) reflection. The instrumental line broadening $\beta_{MB}$ was 4.538 × 10^−3^ rad for the reflection at 2θ~17° and 6.685 × 10^−3^ rad for the reflection at 2θ~30°.

θ is the Bragg angle and β was calculated from the fitting related to (*hkl*) reflection with the pseudo-Voigt cross function using OriginLab2019.

The interlayer spacing of the reflection planes can be calculated from the Bragg’s law as follows:

(6) $$d_{\mathrm{hkl}}=\frac{n\cdot \lambda }{2\cdot \sin\theta }$$

where $d_{hkl}$ is the interlayer spacing of the (*hkl*) lattice planes, λ is the wavelength of the incident wave, and θ is the scattering angle.

The average number of planes N in the crystalline domain can be calculated as follows:

(7) $$N=\frac{L_{hkl}}{d_{hkl}}+1$$

From the corrected azimuthal intensity distribution of the equatorial (200) reflection, the relative orientation degree (OD) and the Herman’s orientation factor f*_H_* parallel to the fiber axis were evaluated for all fibers. The azimuthal intensity distribution of the equatorial reflection at 2θ~17° was corrected by the underground from the meridional scan.

The relative orientation degree can be calculated from the corrected plot of the azimuthal intensity distribution as follows:

(8) $$OD=\;\frac{180{}^{\circ}\;-\;FWHM_{az\left(200\right)}}{180{}^{\circ}}$$

The term $FWHM_{az\left(200\right)}$ is the full width at half intensity maximum of the corrected azimuthal intensity of the reflection at 2θ~17° and can be calculated from a suitable fit function, that is, Pearson VII. The fibers were fully oriented if OD was near to 1 and non-oriented if OD was near to 0. The Herman’s orientation factor was calculated from the following equations:

(9) $$f_{H}=1\;-\;\frac{3\bar{sin^{2}\phi }}{2}$$

and

(10) $$\bar{sin^{2}\phi }=\frac{\int_{0}^{\frac{\pi }{2}}I\left(\phi \right)sin^{2}\left(\phi \right)\cos\left(\phi \right)d\phi }{\int_{0}^{\frac{\pi }{2}}I\left(\phi \right)\cos\left(\phi \right)d\phi }$$

The crystals in the fiber structure were perfectly oriented alongside the fiber axis if $f_{H}$ was near to 1 and random for $f_{H}=0$.

If $f_{H}$ was negative or near to −0.5, this means the crystals were perpendicular or perfectly perpendicular, respectively, to the symmetry axis of the fibers.

#### 2.2.6. Thermogravimetric Analysis (TGA)

The TGA measurements were performed using the TGA Q5000 from TA Instruments (Water Corporation, New Castle, USA) under nitrogen and air. The initial sample weight was ~6 mg and the gas flow was adjusted to be 50 mL·min^−1^. The measurements were performed under the heating rates of 5, 10, 20, and 50 K·min^−1^.

#### 2.2.7. Shrinkage Measurements

Shrinkage measurements were performed on bundles of precursor fibers consisting of 20 single filaments using DMA Q800 from TA Instruments (Water Corporation, New Castle, USA) at an initial gauge length of 16.20 mm. The force resolution of the device was 0.01 mN, the displacement resolution was 1 nm, and the sensitivity of the loss factor tan δ was 0.0001. The shrinkage tests at different pre loads and different heating rates (1, 2, and 5 K·min^−1^) were performed on a bundle of 20 filaments in the temperature range between 50 and 325 °C to understand in-depth the impact of lignin oligomers on the kinetics of the heat-induced physical entropic shrinkage and on the kinetics of the chemically induced shrinkage during the oxidative stabilization at elevated temperatures under air (oxidative environment) under different loads.

#### 2.2.8. Differential Scanning Calorimetry (DSC)

DSC measurements under nitrogen and under air were conducted for the regenerated neat PAN and PAN/lignin fibers using Discovery DSC 2500 from TA Instruments (TA Instruments (Water Corporation, New Castle, USA) with Fusion Cell^TM^ technology for flat baseline. The stabilization procedures were performed under air to mimic the stabilization process and under nitrogen to reveal the stabilizing role of lignin on the stabilization process of the developed fibers. The weight of the samples was between ~3 and 3.5 mg. All samples were pre-dried under vacuum for 24 at 80 °C and stored in a sealed desiccator over silica gel pellets prior to any measurement. Heating rates of 1, 2, 5, 7, and 10 K·min^−1^ were selected for the DSC measurements.

#### 2.2.9. Scanning Electron Microscopy (SEM) and Energy-Dispersive X-Ray Spectroscopy (EDX)

For morphology and structure evaluation of the fibers, SEM analyses were carried out on the highly stretched fibers using a high resolution CrossBeam^®^ NEON^®^ 40 EsB system with FEG (Field Emission Gun) from Carl Zeiss Microscopy GmbH, Germany and an SEM ULTRA plus with FEG from Carl Zeiss Microscopy GmbH, Germany, and equipped with energy-dispersive X-ray spectroscopy (EDX) XFlash^®^ FlatQUAD Detector from Bruker Nano GmbH, Germany.

SEM of the fiber surface and cross sections as well as EDX scans of the cross section of the highly stretched fibers embedded in epoxy were performed to reveal topographic information and the existence of chemical elements in the fiber structure. It was important to maintain that the detection threshold of the elements was given by 0.1 wt. %. Any traces under this limit were not able to be detected using this method. SEM images were taken at 3 keV and EDX measurements at 6 keV primary beam energy. All samples were coated with carbon prior to analysis with SEM and EDX to prevent charging artefacts.

#### 2.2.10. Microscopy

For the evaluation of fiber diameter and cross section of the fibers, the fibers were embedded in an epoxy resin and polished to 0.1 µm. The polished surfaces were then investigated using (VHX 100, Keyence Corporation, Osaka, Japan) polarized reflected-light microscope with magnification up to 2000×.

## 3. Results and Discussions

### 3.1. Micro-Compounding

Table 1 shows the composition of the three investigated dopes, which were prepared utilizing the micro-compounder DSM MC-15.

**Figure 1:**
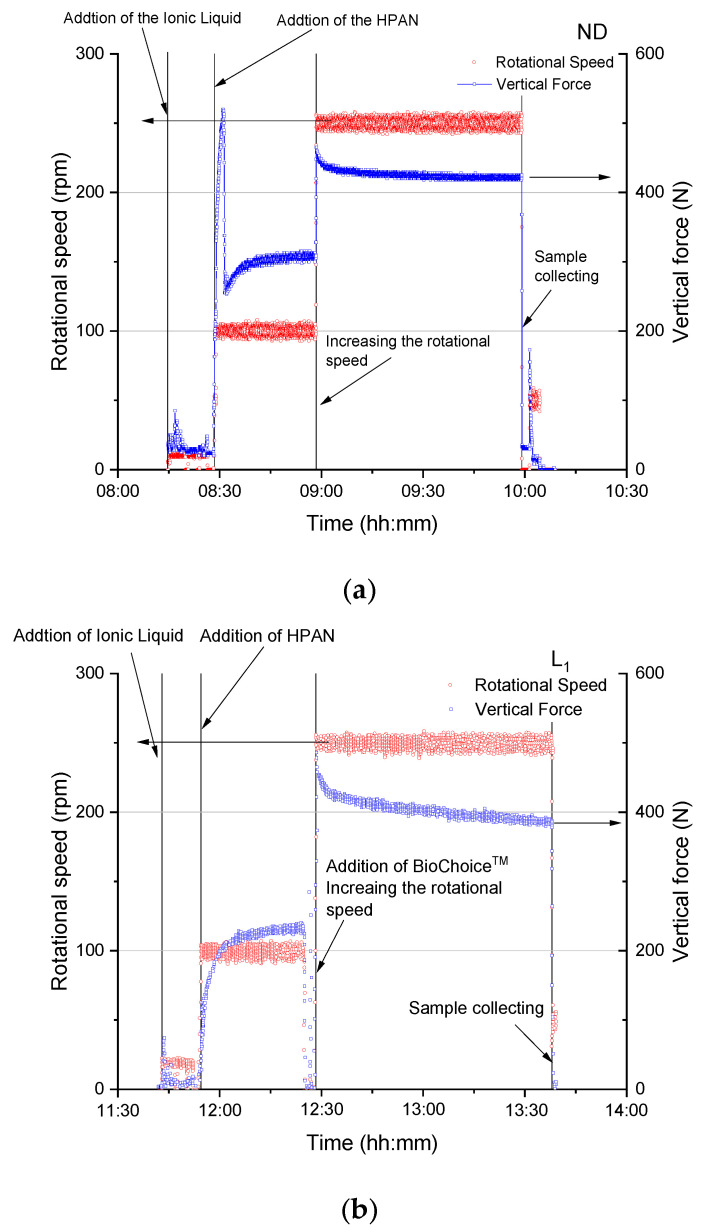
Compounding procedure, rotational speed and dwell time of the (**a**) PAN/IL dopes (ND) and (**b**) PAN/lignin/IL (PAN/lignin = 2:1) (L1) and the impact of the added lignin on the vertical force of the compounding screws. HPAN, homo-polyacrylonitrile.

Figure 1 shows the procedure of the preparation of the dopes ND and L1 and the impact of lignin on the reduction of the axial force during the compounding.

All dopes show relaxation behavior after the increase of the compounding speed from 100 to 250 rpm and holding the speed at 250 rpm. For lignin samples, the relaxation rate was higher than that without lignin, implying the softening effect of the lignin oligomers during compounding. This could be induced by the low molecular weight of lignin.

### 3.2. Rheological Behavior

**Figure 2:**
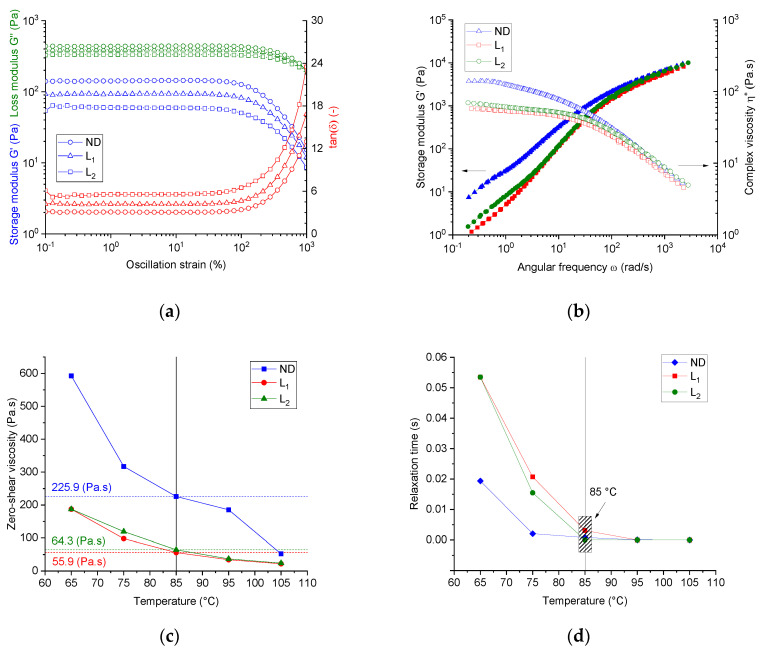
(**a**) Deformation sweep test of the spinning dopes at 85 °C; (**b**) master curves of the complex viscosity versus the angular frequency of the spinning dopes at a reference temperature of 85 °C; (**c**) plot of the zero-shear viscosity versus the temperature for all studied spinning dopes; (**d**) plot of relaxation time of the dopes versus temperature.

Figure 2a shows the linear viscoelastic (LVE) range of the three spinning dopes at 85 °C. The system ND (PAN/IL) showed the lowest critical deformation strain of 16.6%. Spinning dopes with lignin show higher critical deformation strains, which are enhanced with the increasing lignin content, and the values of the critical deformation strain are 21.4% and 27.7% for the dope L1 and L2, respectively. These results suggest that the presence of lignin promotes the resistance of these dopes to deformation, regardless of the low molecular weight of lignin.

In the LVE region, the loss modulus was greater than the storage modulus for all dopes (PAN/IL and PAN/lignin/IL) in the entire deformation range, indicating a dominant viscoelastic liquid-like behavior of all spinning dopes.

It was also clear that the lignin-free spinning dope (ND) showed a higher storage modulus in the LVE region than the system L1. With any further increase in the lignin content, a decrement of the storage modulus value can be found in the LVE region. The same behavior was observed for the loss modulus. This implies that lignin acts as a softening agent in the dopes owing to the low molecular weight of lignin.

For further oscillatory investigations, a deformation strain of 6% was adjusted, thus this value was high enough concerning the rotational moment resolution of the utilized rheometer and fulfils the criteria of the LVE region for all dopes.

The storage modulus G′ and complex viscosity $\eta^{*}$ versus the angular frequency ω as a log–log plot obtained from the frequency sweep tests are illustrated in Supplementary Data Part II (Figure S1). All spinning dopes show strong dependence of the complex viscosity on the temperature, and a general shear thinning behavior at increasing angular frequency and increasing temperatures was detected.

In the terminal region (at low angular frequencies), linear polymer melts typically show the response of a viscoelastic liquid with the scaling property of approximately G′ ∝ ω^2^ and G″ ∝ ω, indicating a liquid-like behavior.

In the case of the pseudo-molten spinning dopes, at, for example, 85 °C, however, the PAN/IL (ND) dope showed a slope of G′ versus the angular frequency ω of 1.04 and a slope of G″ versus ω of 0.76 at small terminal values of ω. The dope L1 (PAN/lignin is 2:1) showed corresponding slopes of 1.36 (G′ versus ω) and 0.95 (G″ versus ω), while the dope L2 (PAN/lignin was 1:1) showed slopes of 1.03 and 0.92, respectively.

The storage and loss moduli of the dopes show, in general, frequency dependency, and are thus considered to be viscoelastic, although the scaling properties are different from polymer melt, where G′ ∝ ω^2^ and G″ ∝ ω.

Adding lignin in small concentrations, that is, dope L1, leads to an increase of both the slope of G′ versus ω and the slope of G″ versus ω in comparison with the case of neat PAN dopes (ND). For dope L1, the increase of the slope of G′ versus ω was higher than the increase of G″ versus ω, implying that lignin oligomers promote more elasticity to the dopes. This suggests, furthermore, some molecular interaction between the PAN and lignin oligomers. Further increase of the share of lignin in the blend, that is, the case of dope L2, however, reduces the slope to values comparable of that of PAN. This suggests the softening effect of lignin at high lignin shares.

From the frequency sweep tests, the master curves of the complex viscosity versus the angular frequency with a reference temperature of 85 °C, for all dope systems, can be seen from Figure 2b. By assuming the applicability of the Cox–Merz rule (|η* (ω)|→η($\dot{\gamma }$)) for the developed dopes, the steady shear viscosity as a function of the shear rate $\dot{\gamma }$ can be calculated. All dopes showed a temperature-dependent shear-thinning behavior.

Low molecular lignin reduced the zero-rate viscosity, that is, in the case of dope L1. However, when increasing the lignin share, that is, dope L2, a slight increase of the zero-shear viscosity over that of dope L1 was observed, obviously owing to the increased overall polymer content in the dope. From the transformed curves of the steady shear viscosity versus the shear rate and applying the Carreau–Yasuda fit function, the specific temperature-dependent and composition-dependent characteristics of all the dopes, that is, the zero-rate viscosity and relaxation time, were calculated.

Figure 2c shows the dependence of the zero-shear viscosity on temperature for all dopes. In general, the dopes with lignin shares of 33.5 wt. % in the PAN matrix showed lowered zero-shear viscosity over the studied temperature range than that of the neat PAN dopes. Higher lignin shares, that is, dope L2, slightly increased the zero-shear viscosity compared with that of dope L1, owing to the higher overall polymer content in the dopes (please refer to Table 1).

Figure 2d illustrates the influence of the temperature and the composition of the dopes on the calculated relaxation time (the consistency parameter). A higher temperature reduced the relaxation time of all dopes and the neat PAN dopes showed the lowest relaxation times. Introducing lignin to the dopes, that is, dope L1, increases the longest relaxation time of the dopes. This effect became strongly visible at lower softening temperatures <85 °C. This implies that the low molecular weight lignin oligomers considerably decelerate the relaxation process of the PAN chains. This effect is very important to be taken into consideration, as the coagulation process will be performed at much lower temperatures.

The compatibility of the dopes was determined using the Han plot, which is temperature invariant and showed a linear correlation in the plot log G′ versus log G″. The system can be considered as compatible if nearly the same slope of the pure matrix can be observed at different lignin shares in the blend; otherwise, the system was considered to be phase-separated [41].

**Figure 3:**
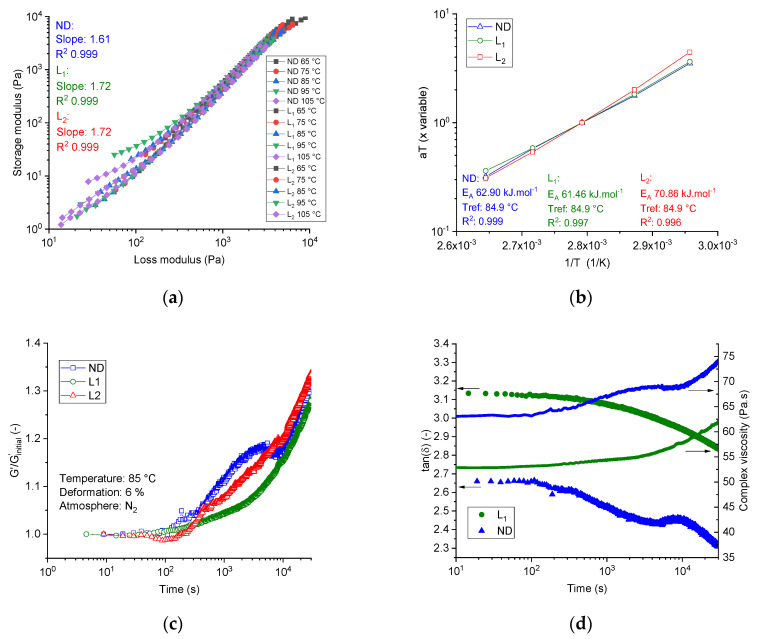
(**a**) Han-plot of the spinning dopes investigated at different temperatures and in the frequency range 0.1–100 Hz; (**b**) time–temperature–superposition (TTS)-Arrhenius plot of the horizontal shift factors versus the temperature at a reference temperature of 85 °C; (**c**) time sweep tests of the developed dopes at 85 °C and 6% deformation; (**d**) development of the loss factor over time at 85 °C from the time sweep tests for the investigated dopes.

Figure 3a shows the Han plot of the three dopes at different temperatures (over the frequency range of 0.1 to 100 Hz). The neat PAN dope (ND) showed a slope of 1.61. Introducing lignin to the dope systems at different lignin shares, that is, dope L1 and L2, slightly increased the slope to 1.72 (+ 6.2%) in both cases. Although lignin consists of low molecular oligomers and showed no repeated monomer sequences, the spinning dopes with different lignin content showed temperature-independent and almost composition-independent slope values of 1.61–1.72, indicating the compatible composition and single-phase consistency between the PAN and low molecular weight purified softwood Kraft lignin BioChoice^TM^.

Additionally, the miscibility/immiscibility of the PAN, lignin, and ionic liquids was also investigated using the time–temperature–superposition plot. The G′(ω) curves for thermo-rheologically simple pseudo-melts were superimposed by horizontal shifts (αT) along the angular frequency axis. The vertical shifts (*b*T) were set to unity. Figure 3b showed the TTS-Arrhenius plot of the three investigated dopes at a reference temperature of 84.9 °C. Regardless of how strong or weak the intra-molecular interactions between lignin oligomers and PAN chains were, the TTS principle was valid for the three spinning dopes. Certainly, lignin oligomers caused no break to the TTS plot at shares up to 50 wt. %, hence the compositions can be miscible.

Isothermal time sweep oscillation tests under nitrogen were used to characterize the thermal stability by observing the development of the nominal value of the storage modulus (G′/G′_Initial_) at 85 °C and 6% deformation over a pre-defined period of time of 30,000 s.

The initial value of the G′_Initial_ was set to 200 ± 10 Pa. The values of the angular frequencies, at which the initial value of G′ maintained the aforementioned values, can be attained from the intercept of the line (G′ = 200 Pa) with the function G′(ω) (Figure 2b). In general, if no molecular changes in the dopes under isothermal conditions take place, the storage modulus should remain constant, and thus time-independent. The system can be, hence, termed as thermally stable. Any decrease in the storage modulus over time will be caused by the molecular degradation affecting the relaxation process, whereas the increase of the storage modulus over the time signifies many molecular changes, for example, cross-linking reaction or gelation. In the case of the filled system, this would mean development in the structural rigidity or elasticity. Figure 3c illustrates the time sweep test of the dopes at 85 °C and a deformation of 6%. After 30,000 s, the neat PAN dopes show an increase of the storage modulus of 32.6% relative to the initial value. Lignin-containing dopes L1 and L2 showed comparatively similar behavior and an increase of the storage modulus by 27.9% and 33.3%, respectively.

Figure 3d shows the development of the loss factor in the time sweep tests at 85 °C over the pre-defined period. Obviously, the neat PAN and lignin-containing dopes show a similar tendency. The loss factors decreased by 11.5% for the neat PAN dope system and by 9% for lignin-based dopes over time, where the increasing rate of the storage modulus was higher than the increasing rate of the loss modulus. This means that the material became, under the applied isothermal conditions over the time, more elastic than viscous. The complex viscosity of the neat PAN system showed an increase of 15%. The complex viscosity of the lignin-containing dopes L1 and L2 increased by 14.6% and 18.7%, respectively, as illustrated in Figure 3d.

### 3.3. Spinning by Dry-Jet Wet Method

The spinning trails were conducted for the dope of neat PAN (ND) and the lignin-containing spinning dope (L1). The spinnability was investigated and the spinnability charts were summarized in Supplementary Data Part III. Depending on the spinnability and the stability in the stretching zone, further investigation matrix was constructed in order to pursue the study of (a) the take up speed at the first godets, (b) the temperature of the coagulation bath temperature and (c) the in-line and post stretching in solid-state on the morphology and fine fiber structure as well as on the mechanical properties of the fibers.

Table 2 summarizes the investigated parameters for both dopes PAN/IL (ND) and PAN/lignin/IL (L1).

**Figure 4:**
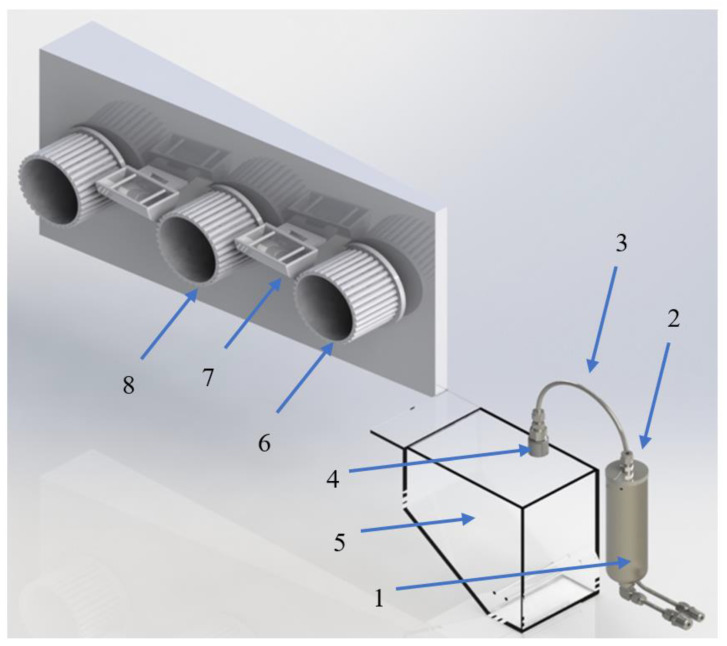
Schematic illustration of the Dry-Jet Wet Spinning set-up: (**1**) spinning dope tank; (**2**) nitrogen inlet; (**3**) spinning dope out-let; (**4**) spinning nozzle; (**5**) coagulation bath; (**6**) first take-up glass godet; (**7**) hot water stretching bath; (**8**) second glass godet.

Figure 4 schematically illustrates the Dry-Jet Wet spinning setup utilized in this work.

### 3.4. Evaluation of the Drawing Dependent Fineness and Diameter

**Figure 5:**
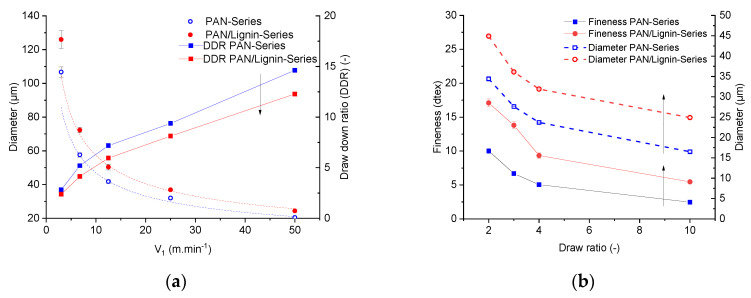
(**a**) The influence of the take-up speed on the diameter of the as-spun PAN and PAN/lignin fibers and on the draw down ratio (DDR). All spinning trails were conducted at 85 °C and a pump rotational speed of 0.5 rpm. (**b**) The influence of the in-line drawing and the solid-state drawing on the fineness and the diameter of the fibers (spinning conditions: V1 = 10 m·min^−1^; T = 85 °C; T_C_Bath_ = 6 °C; pump rotational speed = 0.5 rpm).

Figure 5a showed the influence of the take-up speed of the first godet (V1) on the diameter of the as-spun fibers and on the draw down ratio. Obviously, thicker filaments were obtained from lignin-containing dope in comparison with the neat PAN dope under similar processing conditions (spinning temperature 85 °C; T_C_bath_ = 6 °C).

In the Dry-Jet Wet spinning process, the flow of the extruded mass in the air gap can be considered as a non-isothermal viscoelastic extensional flow assuming a simplified cylindrical symmetry along the spinning line. Ionic liquids have in general low-volatility [42]. Taking the mass-transfer or continuity equation, the momentum equation, the energy equation, and the constitutive equation into consideration, the diameter of the fibers at the first godet is related to the following parameters [43]:

$$D^{2}\propto Q;\;\frac{1}{V_{1}};\;\frac{1}{\rho };c$$

where

D is the diameter of the fiber in µm collected on the first godet,
Q is the mass throughput in g·min^−1^,
ρ is the density of the dope in g·cm^−3^,
c is the polymer fraction in the spinning dope in wt. %,
$V_{1}$ is the take-up speed of the first godet in m·min^−1^.

At a constant take-up speed set-up and the spinning nozzle (D0 = 300 µm), the ratio of the final fiber diameter of lignin-containing fibers D2 to the diameter of the neat PAN fibers D1 was related to the following parameters:

$$\frac{D_{2}^{2}}{D_{1}^{2}}\propto \frac{Q_{2}}{Q_{1}};\;\frac{\rho_{1}}{\rho_{2}};\;\frac{c_{2}}{c_{1}}$$

Figure 5a illustrates higher diameter values of the lignin-containing fibers compared with the diameter values of the neat PAN fibers for a given series of the take-up speed.

The mass throughput of the lignin-containing dopes and the neat PAN was 0.09 g·min^−1^ and 0.11 g·min^−1^, respectively; the polymer content ratio c2/c1 was equal to 18.37/13.04 = 1.41. Taking these parameters into account, the density ratio of the spinning dopes $\rho_{1}/\rho_{2}$ must be greater than 1 in order to analyze the experimental data of the fiber diameter. The dynamic simulation of diameter formation in the Dry-Jet wet spinning of lignin-containing dopes will be conducted in another paper and was not within the scope of the present paper. A discrepancy in the draw down ratio between the neat PAN fibers and PAN/lignin fibers was detected and the discrepancy was increased by increasing the take-up speed. This effect became intense by in-line stretching and solid state post drawing the filaments, as shown in the Figure 5a.

At a low in-line draw ratio (DR = 2), the discrepancy was ca. 30%. At the highest solid-state drawing (DR = 10), the difference was roughly 51%. This means that the draw ratio-dependent decrement of the diameter (lignin-containing fiber) is much lower than that of neat PAN fibers. This effect can also be seen from the values of the linear density.

The fineness of the developed fibers can be seen in Figure 5b. At a total draw ratio of 10, neat PAN fibers showed linear density of 2.46 ± 0.14 dtex and diameter values of 16.5 ± 0.16 µm. Neat PAN fibers were thinner than the lignin-containing fibers 5.46 ± 0.37 dtex with diameter values of 24.9 ± 0.27 µm. From the linear density and the fiber diameter, the theoretically determined density $\rho_{F}$ of the fibers can be calculated from the following equation:

(11) $$\rho_{F}=\frac{4\cdot T_{tex}\cdot 10^{3}}{\pi \cdot D^{2}}$$

where $T_{\mathrm{tex}}$ is the fineness of the fibers in tex, ρ is the density of the fibers in g·cm^−3^, and D is the diameter of the fiber in µm.

The theoretically determined density of highly drawn (DR = 10) neat PAN fibers and highly drawn (DR = 10) lignin-containing fibers was 1.15 g·cm^−3^ and 1.12 g·cm^−3^, respectively.

### 3.5. Mechanical Properties

**Figure 6:**
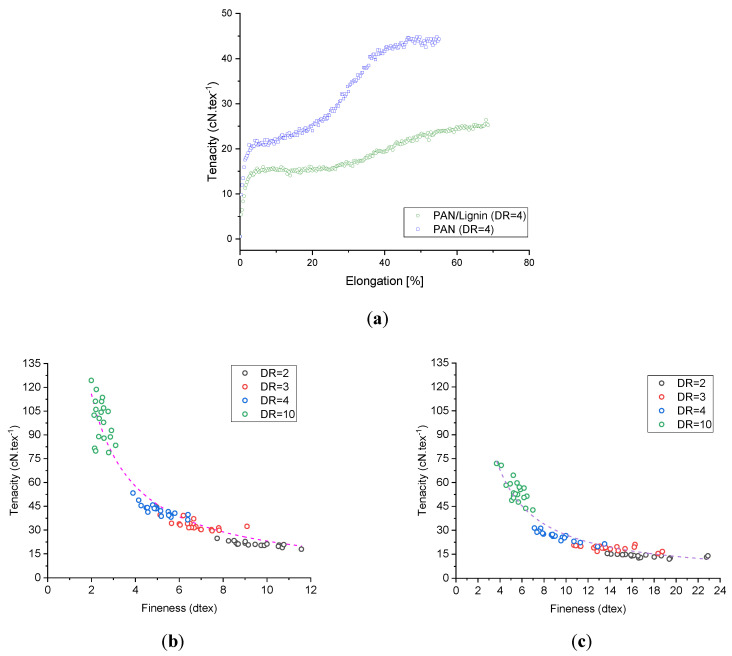
(**a**) Tenacity–elongation curves of PAN and PAN/lignin fibers (draw ratio (DR) = 4; spinning temperature 85 °C; coagulation bath temperature 6 °C; pump rotational speed 0.5 rpm; and V1 = 10 m·min^−1^); tenacity–fineness curves of (**b**) PAN-series and (**c**) PAN/lignin-series at different draw ratios.

The PAN and PAN/lignin fibers show typical tenacity–elongation curves, as shown in Figure 6a. Lignin-containing fibers show lower tenacity values under the same manufacturing conditions. This general tenacity effect is owing to (a) the higher fineness values (lower diameters) of the neat PAN fibers and (b) the disturbed fine structure of the lignin-containing fibers by the low molecular weight lignin oligomers. Nonetheless, lignin-containing fibers in general show higher elongation at break values.

The tenacity of the neat PAN fibers and lignin-containing fibers increases with higher draw ratio, as seen in Figure 6b,c. As it was possible to produce roughly two times finer neat PAN fibers than the lignin-containing fibers under the same conditions (owing to the higher polymer content and lower density of the lignin-containing dopes), better mechanical properties of the neat PAN fibers were reached. Table 3 illustrates in detail the impact of two important processing parameters, that is, the draw ratio and the temperature of the coagulation bath, on the fineness and mechanical performance of the neat PAN and lignin-containing fibers. Obviously, in both cases of neat PAN fibers and lignin-containing fibers coagulated at 6 °C, the tenacity increases with increased drawing. Generally, lignin-containing fibers show lower tenacities and tensile moduli. The highest tenacity of the neat PAN fibers reached an average value of 99.18 ± 2.99 cN·tex^−1^ at a total draw ratio of 10. On the basis of the diameter calculations (D = 16.5 ± 0.16 µm), the tensile strength and the tensile modulus were 1141.04 ± 34.38 MPa and 66.86 ± 3.19 GPa, respectively. These tenacity values obtained from a textile grade PAN by this method were higher than those of many high-performance PAN fibers for CF described in the available literature [44,45,46,47]. For lignin-containing fibers stretched 10 times, the tenacity was 54.96 ± 1.73 cN·tex^−1^. On the basis of the diameter mean value (D = 24.90 ± 0.27 µm) of this fiber series, the tensile strength was 616.24 ± 19.50 MPa and the tensile modulus was 29.89 ± 1.25 GPa. The elongation at break of the neat PAN fibers decreases from 89.34 ± 3.83% for fibers with DR = 2 to 12.73 ± 0.46% for the fibers drawn ten times. Comparatively, lignin-containing fibers showed similar behavior, where the elongation at break reduced from 88.56 ± 4.30% to 13.27 ± 0.41% by increasing the draw ratio from 2 to 10. This implies that low molecular lignin oligomers do not generally affect the elongation at break of the fibers. Additional work regarding the critical fiber radius and its dependency on the fineness will be conducted in the future to understand in-depth the impact of lignin on the brittleness of the fiber structure. Furthermore, Table 3 showed the impact of the coagulation bath temperature on the mechanical performance of the developed neat PAN and lignin-containing fibers. Clearly, the higher the coagulation temperature, the thicker the developed fibers offered lower the mechanical properties of the fibers. This tendency was observed for neat PAN fibers as well as for lignin-containing fibers. For instance, in the case of neat PAN fibers drawn four times in-line, the tenacity was reduced from 41.38 ± 1.88 cN·tex^−1^ to 24.57 ± 0.96 cN·tex^−1^ (reduction by ca. 68.4%). For the lignin-containing fibers, the reduction was roughly 38.1% from 26.13 ± 0.68 cN·tex^−1^ to 18.92 ± 0.55 cN·tex^−1^. This important effect can be explained by the influence of the diffusion rate of the ionic liquids from the high concentration region (the attenuated fiber being drawn in the coagulation zone) to the low-concentration region (the deionized water in the coagulation bath) being higher at higher temperatures. This shortens in turn the available period to draw and thus orient the polymer chains. This was also obvious in terms of higher fineness values and lower orientation parameters (please refer to the crystallographic fine structure section). The orientation is furthermore only possible if the ionic liquids can act as long as possible as a “lubricant-agent” in the fiber structure while orienting the fibers via the extensional forces being applied in the coagulation zone. Another important parameter, which influences the orientation, is the temperature-dependent relaxation time of the spinning dope. At higher temperatures, the dopes, in general, show shorter relaxation times than at lower temperatures. This was of rather importance in the entry zone (after the air-gap) of the attenuated fibers in the coagulation bath. This effect together with the influence of the thermodynamic diffusion rate can be pursued in detail by the orientation values of the fibers, that is, the relative orientation degree and Herman’s orientation factor, as described in the following section. A similar effect of the relaxation time and temperature-dependent diffusion rate of the ionic liquids on the mechanical properties was found in the study of the Dry-Jet Wet spinning of cellulose fibers from ionic liquid dopes [48].

### 3.6. Crystallographic Fine Structure

The structure–process–property relations of the developed fibers were influenced to a wide extent by (a) the material composition, that is, low molecular lignin oligomers, on the deformation behavior of the fibers; and (b) the processing parameters, that is, the coagulation bath temperature, and in-line and solid state stretching. It is well known that the mechanical performance and deformation behavior of the fibers are influenced to a wide extent by the fine structure of the fibers, that is, the crystal structure and the orientation parameters of the crystalline domains. For each type of fiber, equatorial and meridional scans were performed at different drawing ratios and at different coagulation bath temperatures. The azimuthal scans of the intensity of the PAN reflection at 2θ~17° were recorded.

**Figure 7:**
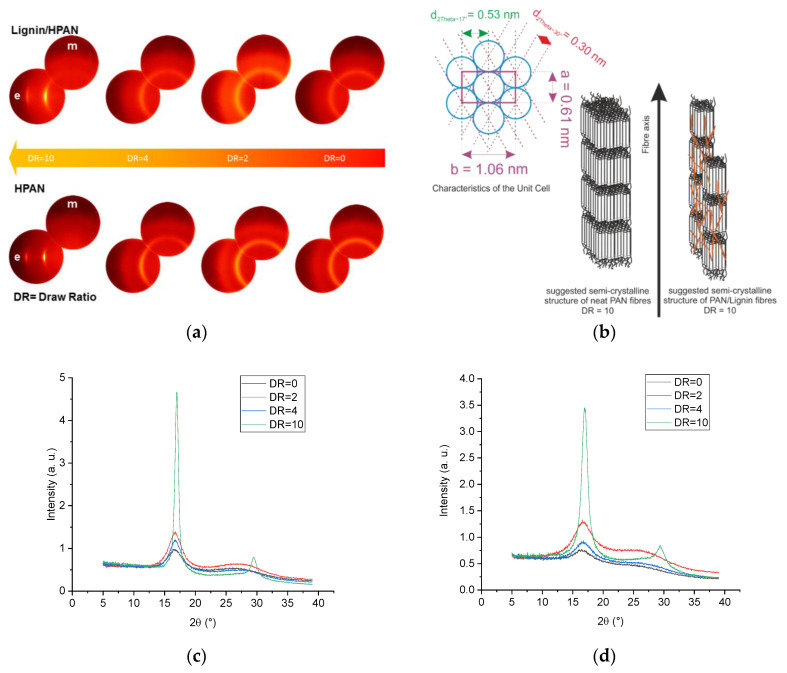
(**a**) Schematic illustration of the 2D equatorial and meridional WAXD patterns of the regenerated PAN fiber series and PAN/lignin fiber series and its development upon the drawing progress; “e” states for equatorial direction and “m” is for meridional direction; (**b**) schematic illustration of the general characteristics of the Unit Cell and the suggested semi-crystalline structure of neat PAN and lignin-containing fibers at DR = 10; equatorial WAXD curves from the radial scans of the equatorial direction of (**c**) the PAN-fiber series and (**d**) PAN/lignin-fiber series.

Figure 7a shows the equatorial (e) and meridional (m) wide-angle X-ray diffraction patterns of the as-spun fibers, in-line drawn fibers, and solid-state post drawn fibers. As-spun neat PAN and lignin-containing fibers (DR = 0) and in-line drawn fibers (DR to 4) show diffuse reflection at 2θ~17° as well as a broad diffuse scattering maximum 2θ values between 22° and 30°. The peak intensity of the reflection at 2θ~17° in the equatorial mode increased and the full width at half maximum decreased with the increased stretching, as shown in Figure 7c,d. Only at high stretching values (DR = 10), neat PAN and lignin-containing fibers showed the highest intensity of the equatorial reflection at 2θ~17° and an equatorial peak at 2θ values ~30° became clear in both cases (Figure 7a,c,d).

Table 4 summarizes the crystallographic fine structural data of the neat PAN fibers and lignin-containing fibers. Additional information about the impact of some processing parameters was also listed. As lignin consists of low molecular oligomers with no defined semi crystalline structure, the semi-crystalline structure of the lignin-containing fibers can only be originally given by the PAN chains. At low temperatures (6 °C) of the coagulation bath, neat PAN fibers show increased equatorial cryptal size L_2θ~17°(e)_ with increasing in-line drawing. In contrast, the meridional crystal size L_2θ~17°(m)_ decreased with increasing drawing. The crystal size ratio L_2θ~17°(e)_/L_2θ~17°(m)_ increased around 39% at in-line draw ratio of 4. The interlayer spacing d_2θ~17°_ = 5.3 Å of the PAN chains was found to be similar to that of the original powder 5.26 Å at all drawing ratios. At a total drawing ratio of 10, the equatorial crystal size L_2θ~17°(e)_ was 12.83 nm. The equatorial crystal size of the reflection at 2θ~30° L_2θ~30°(e)_ was 7.70 nm and the associated interlayer spacing d_2θ~30°_ was 3.0 Å. The aspect ratio of the unit cell b/a (Figure 7b) was equal to 1.732 and the crystal unit is thus hexagonal [49].

Lignin deteriorated to a wide extent the size of the crystal domains, while the characteristics of the unit cell by PAN were still preserved (Figure 7b). In other words, the interlayer spacing d_2θ~17°_ was 5.3 Å and the d_2θ~30°_ was 3.0 Å, hence the aspect ratio of the unit cell b/a was 1.732. The crystal size of the highly oriented lignin-containing fibers was less than that of PAN at the same draw ratio. At 10 times drawing, the crystal size L_2θ~17°(e)_ was 7.16 nm and L_2θ~30°(e)_ was 4.92 nm. This gave a lower number of reflection plains N_2θ~17°_ = 15 and N_2θ~30°_ = 17 than that of neat PAN fibers (N_2θ~17°_ = 26 and N_2θ~30°_ = 26). This implies that lignin did not affect the unit cell characteristics, but disturbed the number of PAN chains contributed in the crystal domain, as seen illustratively in Figure 7b. By increasing the temperature of the coagulation bath, the crystal size was reduced in both cases of neat PAN and lignin-containing fibers as a result of the fast diffusion of ionic liquids from the extruded fibers in the coagulation bath, as seen in Table 4 in both PAN and PAN/lignin-series. The aspect ratio of the L_2θ~17°(e)_/L_2θ~17°(m)_ at a defined drawing was reduced by increasing the coagulation temperature. For instance, this ratio was reduced from 1.53 for neat PAN fibers drawn at DR = 4 to 1.28 for neat PAN fibers coagulated at 20 °C. This suggests that, at higher diffusion rates of the ionic liquids at the equivalent extensional deformation in the coagulation bath (V1 was 10 m·min^−1^ for both cases), the ionic liquids left the fiber at a very early stage, and the chains became unsoftened and thus less able to be oriented. Lowered crystal size was a result of the less orientation, as seen in the Table 4. The similar effect was found in the case of lignin-containing materials. In summary, it was obvious that lignin does not affect the increasing rate of the ratio L_2θ~17°(e)_/L_2θ~17°(m)_, but deteriorates to a wide extent the crystal size L_2θ~17°_. From the azimuthal distribution of the reflection at 2θ~17°, the relative orientation degree (OD) and the Herman’s orientation factor of all developed fibers were calculated.

As shown in Table 4, OD increased by increasing the drawing ratio. For the fiber series coagulated at 6 °C, the highest relative OD was 94.7% for neat PAN fibers and 88.9 for lignin-containing fibers. This implies that lignin reduced and blocked the ability of the PAN chains to be oriented during stretching. The same effect was also visible in terms of Herman’s orientation effect, which was 0.73 and 0.69 for neat PAN and lignin-containing fibers at DR of 10, respectively.

Increasing the coagulation temperature to 20 °C reduced the orientation degree for both series of the developed fibers compared with those fibers coagulated at 6 °C and at the same in-line draw ratio of 2 and 4, as seen in Table 4. These findings support the explanations of the generally lowered mechanical properties at a higher coagulation temperature discussed in the section of the mechanical properties.

Additional research work was also required to investigate the influence of lignin on the periodical structure length (ordered domain length) of the crystal units parallel to the fibers axis as well as the true void length and tilt angle of the voids with respect to the fiber axis. This can be done using the small angle X-ray scattering and will be discussed in another extended research work.

### 3.7. Thermal and Thermo-Mechanical Properties

#### 3.7.1. TGA Measurements

**Figure 8:**
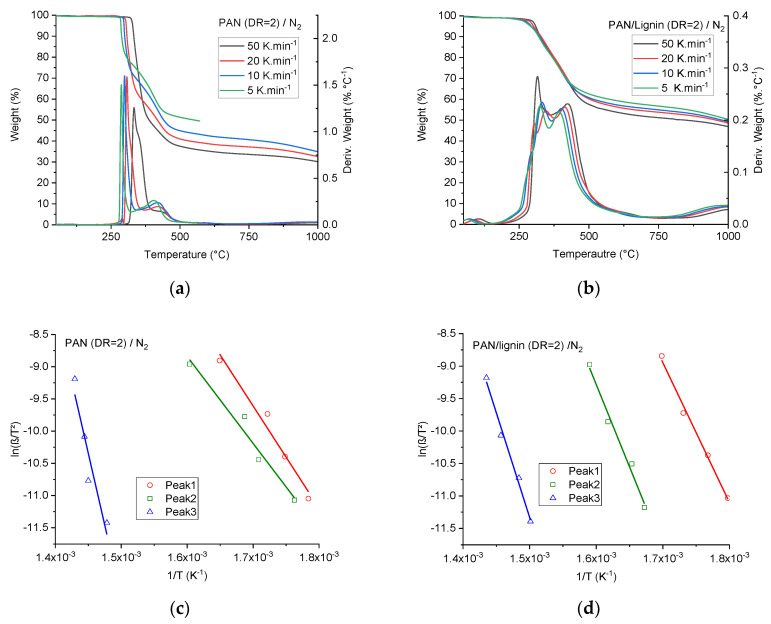
Thermogravimetric analysis (TGA) curves of the (**a**) PAN fibers at a DR of 2 and (**b**) PAN/lignin fibers at a DR of 2 and at different heating rates up to 1000 °C under nitrogen; evaluation of the activation energy of different characteristic thermal degradation processes using the Coats–Redfern method for (**c**) PAN fibers and (**d**) PAN/lignin fibers. For more details about the peak ID assignments, please refer to Supplementary Data Figure S3.

Figure 8a,b show the TGA and DTG curves under nitrogen of the PAN and PAN/lignin fibers (DR = 2), respectively.

Obviously, neat PAN fibers offered lower char residue values than PAN/lignin fibers at a similar heating rate. This implies that PAN/lignin compounds regenerated from ionic liquids show lower thermal degradation rates, and thus possessed higher thermal stability. This could be initiated by cross-linking reactions caused by the ionic liquids. The related DTG curves showed significantly lowered degradation peaks of lignin-containing fibers. The maximum degradation rate was less than 0.3%·K^−1^ within the temperature range between 250 and 500 °C in the case of lignin-containing fibers and around 1.5%·K^−1^ for neat PAN fibers in the same temperature range.

The incorporation of lignin into the PAN system and regeneration from ionic liquids dopes decreased to a wide extent the degradation rate, suggesting that the used softwood lignin participates in and influences to a large extent the degradation and thermally induced cross-linking mechanisms of PAN. Considering that the degradation takes place at DTG values above 0.05%·K^−1^, the degradation of lignin-containing fibers starts significantly at 220 °C and ended at 500 °C under nitrogen. Comparatively, the degradation of neat PAN fibers starts at 250 °C and ceased at around 450 °C.

For a heating rate of 5 K·min^−1^ and at 800 °C under nitrogen, the char residue value of non-stabilized lignin-containing fibers was 56%. This high residue value is important from two points of view:

(a) Neat lignin cannot maintain such values under comparable conditions. Usually, the char residue of lignin lies between 20 and 30% at 800 °C under nitrogen.
(b) For CF, it is important to maintain high carbon yields to increase the productivity, as described in the introduction of this paper.

To further investigate the degradation activation energies from TGA measurements under nitrogen, peak temperatures of different degradation processes were collected at different heating rates. On the basis of these data, the activation energies of different processes were calculated by the Kissinger method from the following plot:

$$ln\left(\frac{\beta }{T_{P}^{2}}\right)versus\;\frac{1}{T_{P}}$$

The activation energy of the related process was then calculated from the slope by the following equation:

(12) $$-\frac{E_{a}}{R}=\frac{d\left(ln\left(\frac{\beta }{T_{P}^{2}}\right)\right)}{d\left(\frac{1}{T_{P}}\right)}$$

where

β is the heating rate in K·min^−1^,
R is the ideal gas constant R = 8.3144598 J·K^−1^·mol^−1^,
$T_{P}$ is the temperature at the peak maximum in K,

From the intercept, the pre-exponential factor A can be calculated. The rate constant k_Tm_ at a defined temperature T_m_ can be calculated from the following equation:

(13) $$k_{T_{m}}=A\cdot e^{-\frac{E_{a}}{R\cdot T_{m}}}$$

For comparison purposes, the activation energy was also calculated using the Flynn–Wall–Ozawa (FWO) method from the following plot:

$$log\left(\beta \right)versus\;\frac{1}{T_{P}}$$

The activation energy of the related process was then calculated from the slope by the following equation:

(14) $$-\frac{E_{a}}{R}=\frac{1}{0.4567}\cdot \frac{d\left(log\left(\beta \right)\right)}{d\left(\frac{1}{T_{P}}\right)}$$

Only degradation peaks from DTG curves greater than 0.05%·K^−1^ of neat PAN and PAN/lignin fibers under nitrogen were considered for this evaluation (Figure 8c,d). The activation energies were listed in Table 5.

Neat PAN fibers showed three main degradation processes, as seen in the Figure 8c,d. This thermally induced degradation behavior is comparable to PAN fibers formed via the wet spinning technique, as described elsewhere [50,51].

For neat PAN fibers, the first degradation under nitrogen is very fast and had a maximum of around 1.5%·K^−1^ with the activation energy of 132 ± 16 kJ·mol^−1^. In conjunction with the following DSC measurements, in this degradation zone, the conversion from nitrile groups to -C≡N to imine groups –C = N- could cyclize in the same chain with the adjacent nitrile group or crosslinks the polymer chains [52]. This reaction is followed by a consequent degradation shoulder with a lowered degradation rate between 0.75 and 1.00%·K^−1^ in the temperature range between 250 °C and 375 °C. The activation energy of this reaction was found to be 112 ± 15 kJ·mol^−1^ according to the Kissinger method. Intramolecular cross-linking reactions take place at this stage according to Rahaman et al. [52]. In the third degradation process, at higher temperatures, polymer chain scission reactions and gaseous volatile by-products, for example, methane, hydrogen, hydrogen cyanide, ammonia, and tarry molecules, could be ejected by the fibers according to the literature [52,53,54]. The highest activation energy was found to be at this stage and was 364 kJ·mol^−1^ according to the Flynn–Wall–Ozawa method. Introducing lignin in the PAN fibers increased the activation energy of the first (179 kJ·mol^−1^) and the second (211 kJ·mol^−1^) reaction processes and reduced the activation energy at elevated temperatures (268 kJ·mol^−1^). This is owing to the fact that softwood lignin is highly susceptible to any thermal processing, which initiates radical polymerization and increases the molecular weight owing to the cross-linking according to the available literature [18,19,55].

#### 3.7.2. Shrinkage Measurements

During the stabilization process of the polyacrylonitrile fibers, two different structural changes take place in a clear sequence [54]. The first one is the entropic physical shrinkage. The origin of this shrinkage is mainly caused by the amorphous region of the polymer, which showed higher chain mobility than the crystalline region. Polyacrylonitrile showed single-hybrid-phase morphology [56,57]. For moderately drawn fibers, the morphology consists of ordered domains with their own glass transition region and amorphous disordered domains with a lower glass transition region [49,56,58,59]. The second part of the heat treatment induced shrinkage of the polyacrylonitrile fibers is a chemical shrinkage, which is mainly caused by the stabilization reactions and the conversion reaction of the nitrile groups -C≡N to imine groups –C = N-, which crosslinks the intra-molecular structure and forms a shorter ladder structure. The crosslinking reactions presume an oxidative environment [54]. Under a constant tensile load of 2.03 mN·tex^−1^, the shrinkage of the fibers was recorded at different heating rates to understand the kinetics of the shrinkage process. The tensile load was generated in the case of neat PAN fibers by applying 0.01 N for 20 filaments of the mean fineness 4.92 tex and by 0.022 N for 20 filaments of the mean fineness 10.94 tex in the case of PAN/lignin fibers, respectively.

**Figure 9:**
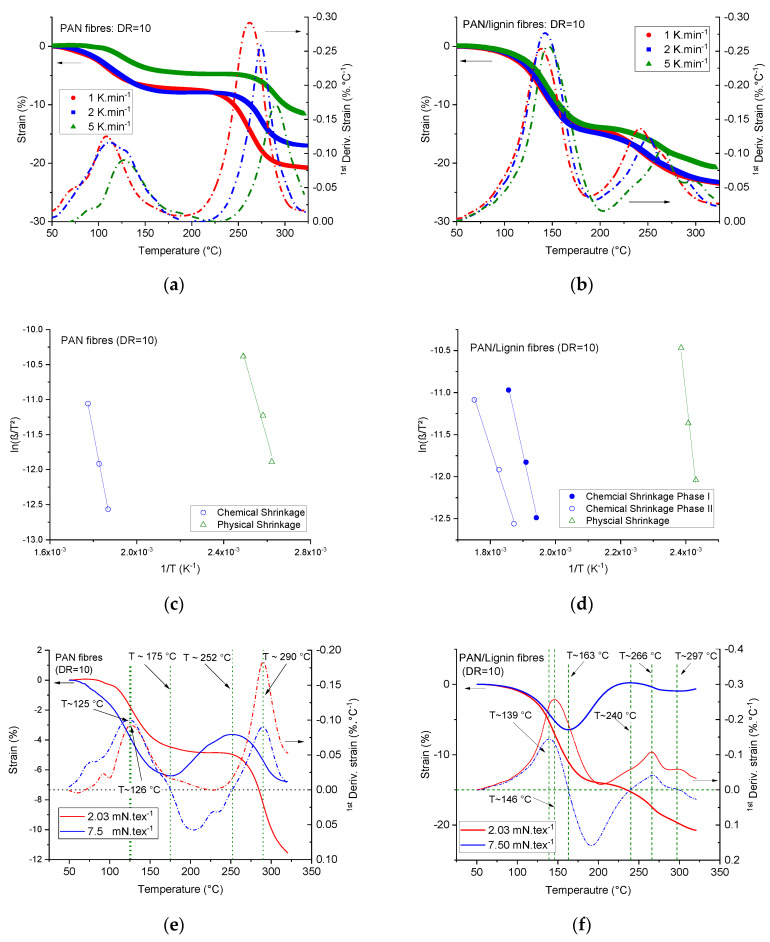
Plot of the strain and first derivative of the strain versus the temperature of (**a**) neat PAN fibers at DR = 10 and (**b**) PAN/lignin fibers at DR = 10 at a specific constant load of 2.03 mN·tex^−1^; Kissinger plot of the physical and chemical shrinkage of (**c**) neat PAN fibers and (**d**) PAN/lignin fibers; impact of the specific pre-tension on the shrinkage behavior of (**e**) PAN fibers (DR = 10) and (**f**) PAN/lignin fibers (DR = 10) recorded at 5 K⋅min^−1^ under air.

Figure 9a,b illustrates the longitudinal shrinkage behavior of the fibers and the first derivative of the strain versus the temperature at different heating rates and at an adequate low specific tension force for both fibers. Neat PAN fibers show in general lower overall shrinkage compared with the lignin-containing fibers. At very low heating rate of 1 K·min^−1^ under air, the overall shrinkage was 20.8% and 23.5 for the neat PAN fibers and lignin-containing fibers, respectively.

For the highly drawn PAN fibers, the chemical shrinkage showed a higher contribution to the overall shrinkage than the physical shrinkage. At a heating rate of 2 K·min^−1^, the chemical shrinkage contributed 53.6% to the overall shrinkage. In contrast, the physical shrinkage of lignin-containing fibers contributed 58.1% (@ 1 K·min^−1^), 63.3% (@ 2 K·min^−1^), and 72.3% (@ 5 K·min^−1^) to the overall shrinkage. This phenomenon is owed to the fact that, the higher the heating rate, the lower the period given to a material to allow it to cross-link and oxidize during the heat treatment.

PAN fibers (DR = 10) showed an activation energy of the physical shrinkage of 92 ± 14 kJ·mol^−1^ (Figure 9c, Table 6). Introducing lignin to the fibers increased the activation energy of the physical shrinkage by around 3.14 times to 289 ± 28 kJ·mol^−1^ (Figure 9d, Table 6). From the crystallographic analysis, lignin decreased the crystalline domain size and increased the amorphous volume part in the fiber structure. This strengthened the assumption of a new amorphous phase of PAN with a higher volume percentage. Knowing that the activation energy is a specific value and represents per definition the minimum amount of energy required to initiate a process or reaction, some new amorphous phase physically bounded or crosslinked to lignin oligomers can be assumed to be responsible for such behavior. Lignin-containing fibers showed furthermore two associated chemical shrinkage phases (Figure 9b). The first chemical shrinkage phase (phase I) of PAN/lignin fibers showed a higher activation energy compared with that of PAN fibers, which were 147 ± 11 kJ·mol^−1^ and 135 ± 3 kJ·mol^−1^, respectively. Phase I is mostly caused by conversion reactions of the nitrile groups -C≡N to –C = N- groups and the intra-chain crosslinking reactions in the ordered domains of the PAN phase. The activation energy of the second shrinkage phase (phase II) was 100 ± 7 kJ·mol^−1^. This shrinkage is assumed to be caused by the cross-linking reactions of lignin. With the increase of the specific tension by 3.7 times, as seen from Figure 9e,f, a reduction of the overall shrinkage of the neat PAN fibers at a heating rate of 5 K·min^−1^ at 325 °C under air from 11.6% to 6.8% can be observed. Obviously, the peak maximum (from the first derivative of the strains versus the temperature) of the chemical shrinkage was found to be load-independent for the studied cases and was at 290 °C. The peak position of the physical shrinkage was shifted to a lower temperature. This change was very small for the studied load cases.

Under a specific tension of 7.5 mN·tex^−1^, PAN fibers started to be extended at 175 °C. This assumes that, at this temperature, the glass transition temperature of some amorphous part of the fibers was reached and the fibers became deformable at the applied tension load. This phenomenon was not detectable at lower tension loads (2.03 mN·tex^−1^). This extension took place over a range of 175 °C to 252 °C, at which the chemical shrinkage started to occur, as illustrated in Figure 9e.

In the case of lignin-containing fibers, however, the onset of glass transition was found at 163 °C and ended at 240 °C with a shift to the left side by roughly ~10 K compared with the neat PAN fibers under the same higher specific tension load. Additionally, the higher load remarkably reduced the peak maximum of the physical shrinkage (obtained from the first derivative of the strain) from 146 °C to 139 °C, as seen from Figure 9f. The chemical shrinkage of phase I occurred at a constant temperature at 266 °C for different loads and the first derivative of the strain versus the temperature was negative, meaning that shrinkage took place. The chemical shrinkage of phase II (lignin-induced) was also at 297 °C in both cases. Remarkably, at this temperature positive, values of the first derivative of the strain versus the temperature were recorded, which means that the fibers at this load level started to be stretched again. This could be initiated by the low molecular volatiles being gassed off by lignin molecules during the early pyrolysis stage (starting from 290 °C, as recorded by the TGA curves). These findings suggest for the future work a detailed evaluation of the activation energies of the physical and chemical shrinkage of the fibers at different specific tension loads and higher drawing ratios.

#### 3.7.3. Thermal Stabilization Kinetics

In order to convert the precursor fibers into CF, a critical thermo-oxidative stabilization step is indispensable. In this step, the fibers must be converted into a thermally more stable state. The mechanisms of the thermo-oxidative stabilization of homo-polyacrylonitrile fibers and copolymers of the acrylonitrile were extensively studied in the literature [52,54,60,61].

In summary, the stabilization reactions of the PAN fibers can be described by conversion of the nitrile groups -C≡N to imine groups -C = N-, dehydration reactions of the PAN backbone chain, oxidation reactions (under air atmosphere), and crosslinking reactions, which take place in an overlapped fashion. Different volatiles, for example, H_2_O, CO_2_, CO, HCN, and NH_3_, were reported as a result of the stabilization reaction [52].

**Figure 10:**
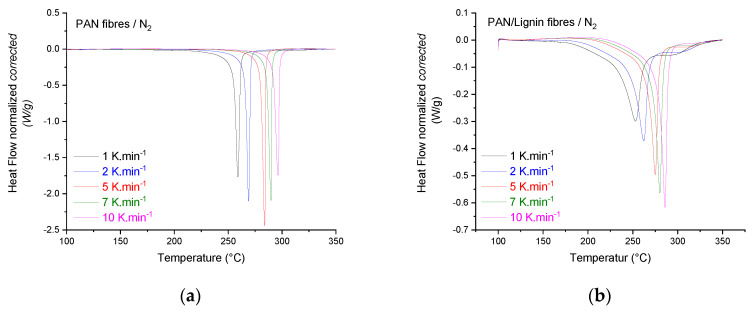

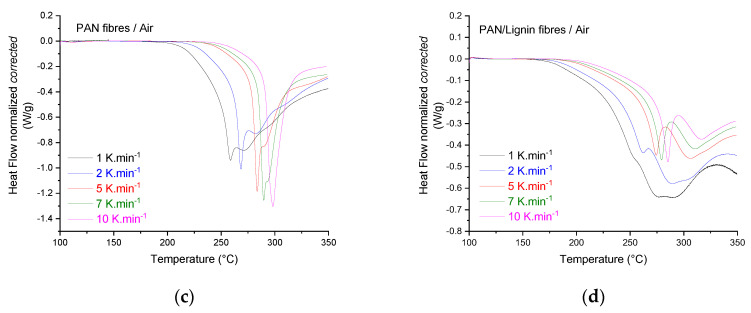
Differential scanning calorimetry (DSC) measurements at different heating rates for (**a**) PAN fibers under nitrogen; (**b**) PAN/lignin fibers under nitrogen; (**c**) PAN fibers under air; and (**d**) PAN/lignin fibers under air.

Figure 10 illustrates the DSC measurements of different heating rates for neat PAN fibers and PAN/lignin fibers conducted under air or nitrogen atmosphere at different heating rates. The measurements were conducted in order to reveal the influence of both oxygen (oxidative air atmosphere) and softwood lignin on the stabilization behavior of the developed fibers.

Under nitrogen (Figure 10a), neat PAN fibers showed single and sharp exothermal peaks of the conversion reaction of nitrile groups to imine groups. This peak shifted to higher temperatures by increasing the heating rate. Introducing lignin to the fibers (Figure 10b) resulted in the overall reduction of the released heat during the exothermal conversion reaction of nitrile groups to imine groups. An additional associated exothermal shoulder was also detected, suggesting exothermal crosslinking reactions induced by lignin. Furthermore, the onset and peak temperatures in the case of lignin-containing fibers shifted (compared with the neat PAN fibers) to lower temperatures by ~10 K for heating rates between 5 and 10 K·min^−1^ and by ~7 K for heating rates between 1 and 2 K·min^−1^.

Neat PAN fibers showed, under oxidative atmosphere (air, Figure 10c), an additional exothermal oxidation shoulder followed by a subsequent exothermal crosslinking reaction peak. Increasing the heating rate reduced the FWHM of the oxidation and crosslinking shoulders and shifted the peaks to higher temperatures. In comparison with the neat fibers stabilized under nitrogen, the peak maxima of the conversion reactions of nitrile groups to imine groups were very slightly shifted to lower temperatures (Figure 10a,c). The addition of lignin reduced the overall released exothermal heat compared with that of neat PAN under the same conditions, suggesting that lignin oligomers actively acted as a stabilizer for the stabilization process.

Utilizing the Kissinger method, the activation energies and the kinetic parameters of the thermal stabilization processes of neat PAN fibers and lignin-containing fibers were calculated under air and nitrogen atmospheres and are summarized in Table 7.

The activation energy of the conversion reactions of the nitrile groups -C≡N to imine groups -C = N- in the case of neat PAN fibers was 146 ± 2 kJ·mol^−1^. This activation energy is comparable—within the linear regression error—to the activation energy of the neat PAN fibers under air, which was 151 ± 5 kJ·mol^−1^. Under oxidative atmosphere, neat PAN fibers showed oxidation and crosslinking reactions with activation energies of 212 ± 29 kJ·mol^−1^ and 117 ± 7 kJ·mol^−1^, respectively. Introducing lignin to the PAN fibers under oxidative atmosphere, the activation energy of the conversion reactions from nitrile to imine groups was increased by 12.30%. Furthermore, the rate constant calculated at 270 °C from the pre-exponential factor A (Table 7) increased from 0.133 to 0.250 min^−1^ by ca. 88%. This implies that lignin oligomers accelerate the conversion reactions of nitrile to imine groups. Comparatively, higher activation energy (163 ± 1 kJ·mol^−1^) of the conversion reaction of nitrile to imine groups was detected for lignin-containing fibers under nitrogen. The rate constant was also increased from 0.124 min^−1^ (under nitrogen) to 0.232 min^−1^. Similar behavior for homo-polyacrylonitrile was reported by Heine et al. [60] at low heating rates up to 10 K·min^−1^. The incorporation of lignin decreased the rate constant at 270 °C of the oxidation reaction by 43.1% from 0.072 to 0.041 min^−1^. The activation energy of the oxidation process was also reduced by 34.1% to 139 ± 60 kJ·mol^−1^. Under air, the constant rate of the crosslinking reaction in the case of the lignin-containing fibers increased by 30%, suggesting the supportive role of lignin to crosslink the structure of PAN. Under nitrogen atmosphere, the activation energy of the crosslinking reaction of PAN/lignin fibers was 236 ± 19 kJ·mol^−1^. This activation energy was reduced by 90% to 124 ± 4 kJ·mol^−1^ for the same fibers under oxidative atmosphere. The constant rate of this reaction was increased 2.43 times from 0.007 to 0.017 min^−1^. Taking the high thermal stability of the lignin-containing fibers into account, which was detected by the TGA measurements, lignin acts furthermore as a stabilizer for the conversion reactions of the nitrile groups to imine groups, supports the crosslinking reactions under air, and inhibits the oxidation reaction of the PAN chains. Additional research work is hereafter of interest to understand the nature of the competitive and/or inhibited reactions caused by softwood lignin incorporation.

### 3.8. Scanning Electron Microscopy (SEM) and Energy-Dispersive X-Ray Spectroscopy (EDX)

**Figure 11:**
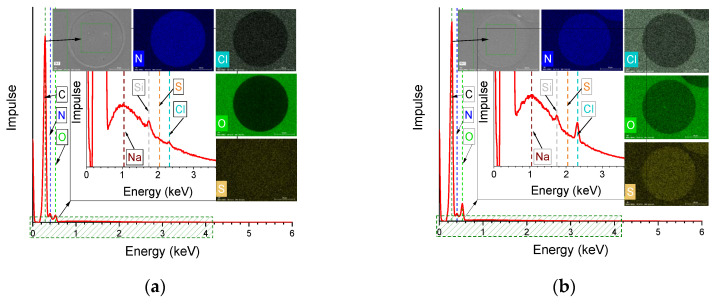

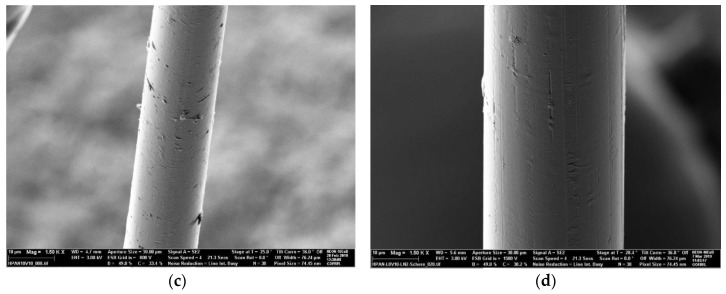
(**a**) EDX analysis of PAN fibers at (DR = 4) and (**b**) EDX curves of PAN/lignin fibers (DR = 4); (**c**) SEM image of the surface of PAN fibers (DR = 10); and (**d**) SEM image of the surface of PAN/lignin fibers (DR = 10).

Figure 11a,b showed the EDX curves of the cross section of the fibers embedded in an epoxy matrix. High resolution images of the SEM of the cross section and the EDX images of the elements nitrogen, chlorine, oxygen, and sulphur are provided in Supplementary Data Figure S3.

The evidence threshold of the atoms lies at 0.1 at. %. Chlorine could not be detected by this method, suggesting the high regeneration rate of the ionic liquids from the fiber structure.

The quantitatively detected portions of C/N/O/S/Cl were 66.17:28.07:5.72:0.03:0.01 in at. %, respectively. The amount of detected chlorine traces was around 0.01 at. %, suggesting a high regeneration rate of the ionic liquids out of the fiber structure. In the case of PAN/lignin fibers, more sulfur (0.16 at. %) was detected (Figure 11b) owing to the chemically bounded sulfur to the lignin caused by the early pulping process. The quantitatively detected portions of C/N/O/S/Cl were 64.99:22.31:12.53:0.16:0.00 in wt. %, respectively.

SEM images (Figure 11c,d) of the fiber surfaces showed, in both cases (PAN and PAN/lignin fibers), smooth and homogenous surface with longitudinal grooves caused by the flow lines in the nozzle during the extrusion process. Neither pores over the cross section of the fibers could be detected nor did a lobulated surface occur, suggesting a controllable and homogeneous coagulation process.

## 4. Conclusions

In the present study, we explored the shear rheological flow behavior, as well as the miscibility, compatibility, and rheological determined thermal stability, of the mixture of PAN, purified softwood lignin, and 1-ethyl-3-methylimidazolium chloride. All investigated dope systems were found to be miscible in term of the Arrhenius plot and compatible for different lignin content in terms of the temperature-invariant Han plot.

The developed fiber based on PAN and lignin offered homogeneous and smooth-surface fibers with fineness values ~6 dtex, tensile strengths ~616 MPa, tensile moduli ~30 GPa, as well as elongation at break ~13.27%.

The coagulation process and coagulation bath temperature extensively influenced the fine crystallographic structure of the fibers. Lowered crystal size, orientation degree, and Herman’s orientation factors were a result of increased coagulation temperatures. No spinning was possible at high coagulation temperatures, that is, at 40 °C. This was found to be correlated to the temperature-dependent diffusion rate of the ionic liquids from the fiber structure in the coagulation bath and to the temperature-dependent relaxation times of the spinning dope. At low coagulation temperatures of 6 °C, lignin was found to deteriorate the crystal size of the PAN phase in the fiber structure while maintaining the dimensions of the unit cell of the PAN phase. Furthermore, introducing the amorphous low molecular weight lignin oligomers to the fibers reduced the orientation degree and Herman’s orientation factors at a given coagulation bath temperature and draw ratio. The essential thermal properties of the developed fibers were investigated. The TGA analysis of the regenerated fibers under inert atmosphere showed unusual and better thermal stability of the lignin-containing fibers than the neat PAN fibers and powders as well as neat lignin powder. This could also be detected at the lowered DTG peaks under the same conditions (heat rate, atmosphere, and temperature range) in comparison with the neat PAN fibers. Lignin increased the activation energy of the characteristic first and second decomposition peaks and lowered the activation energy of the thermal degradation at higher temperatures. The thermo-mechanical shrinkage tests under air supported the findings in the crystallographic fine structure of the fibers. Lignin increased the amorphous part in the fiber structure and introduced a physical interaction with the amorphous part. The existence of a single glass transition of the fibers with a slight positive shift was found in the case of the PAN fibers. Additional research work will be conducted in the future to understand the impact of lignin on the domain length of the PAN phase in the neat PAN fibers and lignin-containing fibers using the small angle X-ray scattering technique. In order to investigate the degree of crystallinity, the ultrafast DSC technique is required, as shown by Furushima et al. in [62]. The crystal conversion mechanisms, orientation recovery mechanisms, and structure formation of the subsequent CF on the base of the developed fibers will also be pursued. It can be also important to mention that no corrosion reactions (caused by the ionic liquids) were found to take place, neither for the measuring geometry of the ARES G2 Rheometer during the long-term rheological characterization nor during the spinning trails.

## Figures and Tables

**Table 1:** Composition and polymer ratios of the investigated dopes in lab-scale. IL, ionic liquid; PAN, polyacrylonitrile.

Sample	IL (g)	PA (g)	Lignin (g)	Mass Ratio PAN/Lignin	Polymer Content (%)	Lignin Share (wt. %)
ND	15	2.25	0	-	13.04	0
L1	15	2.25	1.12	2:1	18.37	33.50
L2	15	2.25	2.25	1:1	23.08	50

**Table 2:** Conducted spinning trails to evaluate the impact of different processing parameters, that is, the temperature of the coagulation bath (T_C_bath_), the take-up speed of the first and the second godets, as well as the off-line drawing on the structure and mechanical properties of the regenerated PAN and PAN/lignin fibers. All spinning trails were conducted at a constant rotational speed of the spinning pump @ 0.5 rpm. *) Post-drawn (2.5 times) in solid-state at 170 °C under air. DR, draw ratio; HPAN, homo-polyacrylonitrile.

Samples		T_C_bath_	V_1_	V_2_	DR
		(°C)	(m·min^−1^)		(-)
PAN/IL	PAN/Lignin/IL	Processing parameters			
Q_1_ = 0.11 (g·min^−1^)	Q_2_ = 0.09 (g·min^−1^)				
HPAN1	HPANL1	6	50		
HPAN2	HPANL2		25		
HPAN3	HPANL3		12.5		
HPAN4	HPANL4		6.75		
HPAN5	HPANL5		3		
HPAN9	HPANL6		10	20	2
HPAN8	HPANL7			30	3
HPAN10	HPANL8			40	4
HPAN10V10	HPANL8V10			40	10 *
HPAN16	HPANL9	20	10	20	2
HPAN17	HPANL10			30	3
HPAN18	HPANL11			40	4

**Table 3:** The impact of the processing parameters (drawing; temperature of the coagulation bath) on the mechanical performance of the developed fibers. Data in brackets represent the standard error. * Data based on the diameter mean values and tenacity mean values.

DR	T_C_bath_	Fineness	Diameter	Tenacity	Tensile Modulus	Elongation at Break	Theoretically Determined Fiber Density	Tensile Strength *	Tensile Modulus *
(-)	(°C)	(dtex)	(µm)	(cN·tex^−1^)	(cN·tex^−1^)	(%)	(g·cm^−3^)	(MPa)	(GPa)
**PAN series**									
2	6	10.01 (0.37)	34.4 (0.25)	20.70 (0.34)	1568.99 (68.73)	89.34 (3.83)	1.08	222.94 (3.66)	16.90 (0.74)
3		6.68 (0.21)	27.6 (0.12)	33.53 (0.86)	2080.00 (156.17)	69.2 (1.73)	1.09	374.37 (9.60)	23.22 (1.74)
4		5.05 (0.14)	23.7 (0.11)	41.38 (1.88)	2863.65 (131.01)	46.36 (2.62)	1.14	473.69 (21.52)	32.78 (1.49)
10		2.46 (0.07)	16.5 (0.16)	99.18 (2.99)	5813.71 (277.50)	12.73 (0.46)	1.15	1141.04 (34.38)	66.86 (3.19)
2	20	11.22 (0.38)	-	17.00 (0.38)	1389.57 (67.65)	74.47 (2.35)	-	-	-
3		8.74 (0.43)	-	27.09 (0.65)	1499.05 (107.30)	73.73 (2.58)	-	-	-
4		10.97 (0.91)	-	24.57 (0.96)	334.82 (25.21)	66.58 (1.76)	-	-	-
**PAN/Lignin series**									
2	6	17.06 (0.56)	44.3 (0.24)	13.94 (0.21)	1098.90 (74.29)	88.56 (4.30)	1.11	154.29 (4.87)	12.16 (1.72)
3		13.83 (0.51)	36.1 (0.16)	18.95 (0.33)	1119.76 (67.97)	86.78 (4.83)	-	256.05 (9.32)	15.13 (1.92)
4		9.34 (0.39)	31.9 (0.23)	26.13 (0.68)	1930.8 (67.86)	66.61 (1.98)	1.16	305.36 (7.95)	22.56 (0.79)
10		5.46 (0.18)	24.9 (0.27)	54.96 (1.73)	2666.13 (111.24)	13.27 (0.41)	1.12	616.24 (19.50)	29.89 (1.25)
2	20	19.72 (1.23)	-	9.72 (0.36)	446.42 (16.70)	70.26 (2.72)	-	-	-
3		19.39 (1.35)	-	16.08 (0.30)	489.94 (6.49)	75.98 (2.19)	-	-	-
4		9.44 (0.39)	-	18.92 (0.55)	257.47 (45.25)	67.43 (2.35)	-	-	-

**Table 4:** Crystal structure parameters of the PAN-fibers and PAN/lignin fibers.

Sample	DR	T_C. Bath_	L_2θ~17°e_	L_2θ~17°m_	L_2θ~17°e_/L_2θ~17°m_	d_2θ~17°m_	L_2θ~30°_	d_2θ~30°e_	N_2θ~17°e_	N_2θ~30°e_	OD	f*_H_*
	(-)	(°C)	(nm)		(-)	(Å)	(nm)	(Å)	(-)	(-)	(%)	(-)
	PAN series											
HPAN3	0	6	2.79	2.54	1.10	5.3	-	-	6	-	32.6	0.51
HPAN9	2		3.05	2.29	1.33	5.3	-	-	7	-	53.3	0.55
HPAN10	4		3.54	2.31	1.53	5.3	-	-	8	-	58.9	0.58
HPAN10V10	10		12.83	-	-	5.2	7.70	3.0	26	26	94.7	0.73
HPAN16	2	20	2.98	2.47	1.21	5.3	-	-	7	-	41.7	0.53
HPAN18	4		3.25	2.54	1.28	5.3	-	-	7	-	50.9	0.54
	PAN/Lignin series											
HPANL3	0	6	1.83	1.81	1.01	5.3	-	-	4	-	14.2	0.50
HPANL6	2		1.97	1.47	1.34	5.3	-	-	5	-	39.9	0.53
HPANL8	4		2.41	1.59	1.52	5.3	-	-	6	-	52.3	0.54
HPANL8V10	10		7.16	-	-	5.2	4.92	3.0	15	17	88.9	0.69
HPANL9	2	20	2.05	1.74	1.18	5.3	-	-	5	-	- *	- *
HPANL11	4		2.13	1.66	1.28	5.3	-	-	5	-	39.6	0.53

* not investigated.

**Table 5:** Activation energy of different degradation peaks of neat PAN fibers and PAN/lignin fibers under nitrogen atmosphere calculated from the Costs–Redfern method and the Flynn–Wall–Ozawa method; * error of the linear regression. For more details about the peak ID assignments, please refer to Supplementary Data Figure S3.

	Kissinger Method					Flynn–Wall–Ozawa Method				
Peak ID	Slope	Error *	Activation energy	Error	R^2^	Slope	Error *	Activation energy	Error	R^2^
	(-)	(-)	(kJ·mol^−1^)		(-)	(-)	(-)	(kJ·mol^−1^)		(-)
PAN series										
1	−15,875.7	±1913.1	132	±16	0.958	−7401.9	±827.1	135	±15	0.963
2	−13,499.5	±1806.6	112	±15	0.948	−6380.1	±782.2	116	±14	0.956
3	−44,665.7	±10,431.7	371	±87	0.852	−19,995.0	±4532.4	364	±82	0.860
PAN/Lignin series										
1′	−21,549.1	±1288.1	179	±11	0.990	−9855.7	±561.3	179	±10	0.990
2′	−25,382.4	±2217.0	211	±18	0.977	−11,556.0	±963.1	210	±17	0.979
3′	−32,221.0	±2357.4	268	±20	0.984	−14,585.1	±1024.6	265	±19	0.985

**Table 6:** Activation energy of the physical and chemical shrinkage of the neat PAN fibers and PAN/lignin fibers calculated from the Kissinger plot at different heating rates under air.

Shrinkage Type	Activation Energy	Error *	R^2^
	(kJ·mol^−1^)	(kJ·mol^−1^)	(-)
PAN series			
Physical	92	±14	0.989
Chemical	135	±3	0.999
PAN/Lignin series			
Physical	289	±28	0.995
Chemical Phase I	146	±11	0.997
Chemical Phase II	100	±7	0.998

**Table 7:** Activation energies and kinetic parameters of the thermal stabilization processes of PAN fibers and PAN/lignin fibers under air and nitrogen. * Error of the linear regression.

Process	Nitrogen					Air				
	Activation Energy	Error *	R^2^	A	k_270 °C_	Activation Energy	Error *	R^2^	A	k_270 °C_
	(kJ·mol^−1^)		(-)	min^−1^	min^−1^	(kJ·mol^−1^)		(-)	min^−1^	min^−1^
PAN series										
Conversion of -C≡N to -C = N-	146	2	0.999	1.49 × 10^13^	0.124	151	5	0.995	4.79 × 10^13^	0.133
Oxidation	-	-	-	-	-	211	28	0.965	1.59 × 10^19^	0.072
Crosslinking	-	-	-	-	-	117	7	0.993	2.55 × 10^9^	0.013
PAN/lignin series										
Conversion of -C≡N to -C = N-	163	1	0.999	1.15 × 10^15^	0.232	170	4	0.998	5.56 × 10^15^	0.250
Oxidation	-	-	-	-	-	139	6	0.993	1.07 × 10^12^	0.041
Crosslinking	236	19	0.975	3.39 × 10^20^	0.007	124	4	0.997	1.35 × 10^10^	0.017

## Supplementary Materials

The following are available online at https://www.mdpi.com/1996-1944/13/17/3687/s1, Figure S1: Frequency sweep tests of (**a**) dope ND; (**b**) dope L1; (**c**) dope L2 at different temperatures., Figure S2: (**a**) Plot of the throughput of the up-scaled spinning dopes ND and L1 versus the rotational speed of the spinning pump; (**b**) the spinnability chart of the spinning dope ND and (**c**) the spinnability chart of the spinning dope L1 (round green circles mean the system was found to be spinnable; and round tacked red circles for un-spinnable set-up. Figure S3: DTG curves of (**a**) PAN fibers and (**b**) PAN/lignin fibers (DR = 2) for detailed assignment of the Peak ID 1, 2 and 3 for both fiber types. Figure S4: SEM Images of (**a**) PAN and (**b**) PAN/Lignin fibres (DR = 2); Green rectangles indicate the areas selected for the EDX evaluations of the elemental concentrations of the atoms C, N, O, S and Cl. EDX images of nitrogen (**c**) and (**d**); oxygen (**e**) and (**f**); sulphur (**g**) and (**h**) and chlorine (**k**) and (**l**). Table S1: Structural properties of Softwood BioChoice^TM^ Kraft Lignin and (*) Softwood Kraft Lignin obtained from the black liquor of Spruce + Pine wood and participated via the LignoBoost^®^ concept.

## References

[1] Al Aiti M., Göbel M., Jehnichen D., Fischer D., Brünig H., Scheffler C., Wulff L., Heinrich G. The Impact of the lattice structure and pore geometry on the mechanical properties of carbon fibers with different tensile modulus (100–950 GPa) studied by Raman spectroscopy, WAXS and SAXS techniques. Proceedings of the SAMP Europe Conference 2017 Stuttgart.

[2] Witten E., Kraus T., Kühnel M. (2016). Composites-Marktbericht 2016: Marktentwicklung, Trends, Ausblicke und Herausforderungen.

[3] Witten E., Sauer M., Kühnel M. (2017). Composites-Marktbericht 2017: Marktentwicklungen, Trends, Ausblicke und Herausforderungen.

[4] Witten E., Mathes V., Sauer M., Kühnel M. (2018). Composites-Marktbericht 2018: Marktentwicklungen, Trends, Ausblicke und Herausforderungen.

[5] Das S., Warren J., West D., Schexnayder S.M. (2016). Global Carbon Fiber Composites Supply Chain Competitiveness Analysis.

[6] Al Aiti M., Jehnichen D., Fischer D., Brünig H., Heinrich G. (2018). On the morphology and structure formation of carbon fibers from polymer precursor systems. Prog. Mater. Sci..

[7] Norris R.E., Kaufman M., Naskar A., Paulauskas F.L., Yarborough K., Derstine C. (2014). Development and Commercialization of Alternative Carbon Fiber Precursors and Conversion Technologies.

[8] Steudle L. (2014). New Precursors for Lignin Based Carbon Fibers.

[9] Horvath B., Peralta P., Frazier C., Peszlen I. (2011). Thermal softening of Transgenic Aspen. Biorescources.

[10] Kubo S., Uraki Y., Sano Y. (1996). Thermomechanical Analysis of Isolated Lignins. Holzforsch. Int. J. Biol. Chem. Phys. Technol. Wood.

[11] Ogale A.A., Zhang M., Jin J. (2016). Recent advances in carbon fibers derived from biobased precursors. J. Appl. Polym. Sci..

[12] Steudle L.M.B., Michael R. (2014). Frank Erik Precursor-Fasern von Lignin-basierten Carbonfasern, deren Herstellung und Verwendung.

[13] Clauss M., Frank E., Buchmeiser M.R. (2015). Verfahren zur Herstellung einer Lignin-basierten Zusammensetzung.

[14] Eckert R.C., Abdullah Z. (2010). Carbon Fibers from Kraft Softwood Lignin. U.S. Patent.

[15] Tomani P. (2010). The lignoboost process. Cellul. Chem. Technol..

[16] Bährle C., Custodis V., Jeschke G., van Bokhoven J.A., Vogel F. (2014). In situ Observation of Radicals and Molecular Products During Lignin Pyrolysis. ChemSusChem.

[17] Wang S., Wang K., Liu Q., Gu Y., Luo Z., Cen K., Fransson T. (2009). Comparison of the pyrolysis behavior of lignins from different tree species. Biotechnol. Adv..

[18] Cui C., Sadeghifar H., Sen S., Argyropoulos D.S. (2013). Toward thermoplastic lignin polymers; Part II: Thermal and polymer characteristics of kraft lignin and derivatives. Biorescources.

[19] Kadla J.F., Kubo S., Venditti R.A., Gilbert R.D., Compere A.L., Griffith W. (2002). Lignin-based carbon fibers for composite fiber applications. Carbon.

[20] Norberg I., Nordström Y., Drougge R., Gellerstedt G., Sjöholm E. (2013). A new method for stabilizing softwood kraft lignin fibers for carbon fiber production. J. Appl. Polym. Sci..

[21] Brodin I., Sjöholm E., Gellerstedt G. (2010). The behavior of kraft lignin during thermal treatment. J. Anal. Appl. Pyrolysis.

[22] Brodin I. (2009). Chemical Properties and Thermal Behaviour of Kraft Lignins.

[23] Ebeling H., Fink H.-P., Lehmann A. (2012). Method for the Production of Lignin-Containing Precursor Fibres and also Carbon Fibres. U.S. Patent.

[24] Ma Y., Asaadi S., Johansson L.-S., Ahvenainen P., Reza M., Alekhina M., Rautkari L., Michud A., Hauru L., Hummel M. (2015). High-Strength Composite Fibers from Cellulose–Lignin Blends Regenerated from Ionic Liquid Solution. ChemSusChem.

[25] Olsson C., Sjöholm E., Reimann A. (2017). Carbon fibres from precursors produced by dry-jet wet-spinning of kraft lignin blended with kraft pulps. Holzforschung.

[26] Bengtsson A., Bengtsson J., Olsson C., Sedin M., Jedvert K., Theliander H., Sjöholm E. (2018). Improved yield of carbon fibres from cellulose and kraft lignin. Holzforschung.

[27] Byrne N., De Silva R., Ma Y., Sixta H., Hummel M. (2018). Enhanced stabilization of cellulose-lignin hybrid filaments for carbon fiber production. Cellulose.

[28] Baker D.A., Rials T.G. (2013). Recent advances in low-cost carbon fiber manufacture from lignin. J. Appl. Polym. Sci..

[29] Klett A.S., Chappell P.V., Thies M.C. (2015). Recovering ultraclean lignins of controlled molecular weight from Kraft black-liquor lignins. Chem. Commun..

[30] Ishikawa N., Uraki Y., Sano Y. (1997). Preparation of lignin fibers from softwood acetic acid lignin: Relationship between fusibility and the chemical structure of lignin. Mokuzai Gakkaishi.

[31] Fields R.P., Ragg L.P. (1990). Production of Low Ash Lignin. U.S. Patent.

[32] Al-Hadithi T.S.R., Barnes H.A., Walters K. (1992). The relationship between the linear (oscillatory) and nonlinear (steady-state) flow properties of a series of polymer and colloidal systems. Colloid Polym. Sci..

[33] Cox W.P., Merz E.H. (1958). Correlation of dynamic and steady flow viscosities. J. Polym. Sci..

[34] Colby R.H. (1989). Breakdown of time-temperature superposition in miscible polymer blends. Polymer.

[35] Ajji A., Choplin L., Prud’Homme R.E. (1991). Rheology of polystyrene/poly(vinyl methyl ether)blends near the phase transition. J. Polym. Sci. Part B Polym. Phys..

[36] Kim J.K., Lee H.H., Son H.W., Han C.D. (1998). Phase Behavior and Rheology of Polystyrene/Poly(α-methylstyrene) and Polystyrene/Poly(vinyl methyl ether) Blend Systems. Macromolecules.

[37] Roovers J., Toporowski P.M. (1992). Microheterogeneity in miscible blends of 1,2-polybutadiene and 1,4-polyisoprene. Macromolecules.

[38] Ngai K.L., Plazek D.J. (1990). Breakdown of time-temperature superposition in miscible polymer blends and the coupling scheme. Macromolecules.

[39] Friedrich C., Schwarzwälder C., Riemann R.E. (1996). Rheological and thermodynamic study of the miscible blend polystyrene/poly(cyclohexyl methacrylate). Polymer.

[40] Pathak J.A., Colby R.H., Kamath S.Y., Kumar S.K., Stadler R. (1998). Rheology of Miscible Blends: SAN and PMMA. Macromolecules.

[41] Han C.D., Kim J. (1987). Rheological technique for determining the order–disorder transition of block copolymers. J. Polym. Sci. Part B Polym. Phys..

[42] Ravula S., Larm N.E., Mottaleb M.A., Heitz M.P., Baker G.A. (2019). Vapor Pressure Mapping of Ionic Liquids and Low-Volatility Fluids Using Graded Isothermal Thermogravimetric Analysis. ChemEngineering.

[43] Xia X., Gong M., Wang C., Wang B., Zhang Y., Wang H. (2015). Dynamic modeling of dry-jet wet spinning of cellulose/[BMIM]Cl solution: Complete deformation in the air-gap region. Cellulose.

[44] Morris E.A., Weisenberger M.C., Bradley S.B., Abdallah M.G., Mecham S.J., Pisipati P., McGrath J.E. (2014). Synthesis, spinning, and properties of very high molecular weight poly(acrylonitrile-co-methyl acrylate) for high performance precursors for carbon fiber. Polymer.

[45] Lyons K.M., Newcomb B.A., McDonald K.J., Chae H.G., Kumar S. (2015). Development of single filament testing procedure for polyacrylonitrile precursor and polyacrylonitrile-based carbon fibers. J. Compos. Mater..

[46] Newcomb B.A., Gulgunje P.V., Gupta K., Kamath M.G., Liu Y., Giannuzzi L.A., Chae H.G., Kumar S. (2015). Processing, structure, and properties of gel spun PAN and PAN/CNT fibers and gel spun PAN based carbon fibers. Polym. Eng. Sci..

[47] Bahl D.P., Mathur R.B., Dhami T.L. (1985). Modification of polyacrylonitrile fibres to make them suitable for conversion into high performance carbon fibres. Mater. Sci. Eng..

[48] Hauru L.K.J., Hummel M., Michud A., Sixta H. (2014). Dry jet-wet spinning of strong cellulose filaments from ionic liquid solution. Cellulose.

[49] Lindenmeyer P.H., Hosemann R. (1963). Application of the Theory of Paracrystals to the Crystal Structure Analysis of Polyacrylonitrile. J. Appl. Phys..

[50] Lee S., Kim J., Ku B.C., Kim J., Joh H.I. (2012). Structural Evolution of Polyacrylonitrile Fibers in Stabilization and Carbonization. Adv. Chem. Eng. Sci..

[51] Xue T.J., McKinney M.A., Wilkie C.A. (1997). The thermal degradation of polyacrylonitrile. Polym. Degrad. Stab..

[52] Rahaman M.S.A., Ismail A.F., Mustafa A. (2007). A review of heat treatment on polyacrylonitrile fiber. Polym. Degrad. Stab..

[53] Edie D.D. (1998). The effect of processing on the structure and properties of carbon fibers. Carbon.

[54] Fitzer E., Frohs W., Heine M. (1986). Optimization of stabilization and carbonization treatment of PAN fibres and structural characterization of the resulting carbon fibres. Carbon.

[55] Sjöholm E., Gellerstedt G., Drougge R., Norberg I. (2014). Method for Stabilizing Lignin Fiber for Further Conversion to Carbon Fiber. U.S. Patent Application.

[56] Bohn C.R., Schaefgen J.R., Statton W.O. (1961). Laterally ordered polymers: Polyacrylonitrile and poly(vinyl trifluoroacetate). J. Polym. Sci..

[57] Hinrichsen G., Orth H. (1971). Direct evidence of colloidal structure on drawn polyacrylonitrile. J. Polym. Sci. Part B Polym. Lett..

[58] Sawai D., Kanamoto T., Yamazaki H., Hisatani K. (2004). Dynamic Mechanical Relaxations in Poly(acrylonitrile) with Different Stereoregularities. Macromolecules.

[59] Colvin B.G., Storr P. (1974). The crystal structure of polyacrylonitrile. Eur. Polym. J..

[60] Heine M. (1988). Optimierung der Reaktionsbedingungen von Thermoplastischen Polymer-Fasern zur Kohlenstofffaser-Herstellung am Beispiel von Polyacrylnitril.

[61] Liu H.C., Chien A.-T., Newcomb B.A., Bakhtiary Davijani A.A., Kumar S. (2016). Stabilization kinetics of gel spun polyacrylonitrile/lignin blend fiber. Carbon.

[62] Furushima Y., Nakada M., Takahashi H., Ishikiriyama K. (2014). Study of melting and crystallization behavior of polyacrylonitrile using ultrafast differential scanning calorimetry. Polymer.

