# Supplementary material for: Dry-Jet Wet Spinning of Thermally Stable Lignin-Textile Grade Polyacrylonitrile Fibers Regenerated from Chloride-Based Ionic Liquids Compounds
Source: Materials (Basel). 2020 Aug 20;13(17):3687. doi: 10.3390/ma13173687 (PMC7504658; doi:10.3390/ma13173687)
Supplement: Supplementary file 1 [file materials-13-03687-s001.pdf]

materials

Supplementary Data

Dry-Jet Wet Spinning of Thermally Stable Lignin-Textile Grade Polyacrylonitrile Fibers Regenerated from Chloride-Based Ionic Liquids Compounds

Muhannad Al Aiti [1,*], Amit Das [2,3], Mikko Kanerva [3], Maija Järventausta [4], Petri Johansson [4], Christina Scheffler [2], Michael Göbel [2], Dieter Jehnichen [2], Harald Brünig [2], Lucas Wulff [1], Susanne Boye [2], Kerstin Arnhold [2], Jurkka Kuusipalo [4], and Gert Heinrich [2,5,*]

1 Institute of Materials Science, Technische Universität Dresden; D-01062 Dresden, Germany; lucaswulff@gmx.de
2 Leibniz-Institut für Polymerforschung Dresden e. V., D-01069 Dresden, Germany; das@ipfdd.de (A.D.); scheffler@ipfdd.de (C.S.); goebel@ipfdd.de (M.G.); djeh@ipfdd.de (D.J.); bruenig@ipfdd.de (H.B.); boye@ipfdd.de (S.B.); Arnholdk@ipfdd.de (K.A.)
3 Polymer Science Engineering, Materials Science and Environmental Engineering, Faculty of Engineering and Natural Sciences, Tampere University, FI-33014 Tampere, Finland; mikko.kanerva@tuni.fi
4 Paper Converting and Packaging Technology, Materials Science and Environmental Engineering, Faculty of Engineering and Natural Sciences, Tampere University, FI-33014 Tampere, Finland; maija.jarventausta@tuni.fi (M.J.); petri.johansson@tuni.fi (P.J.); jurkka.kuusipalo@tuni.fi (J.K.)
5 Institut für Textilmaschinen und Textile Hochleistungsfaserstofftechnik, Technische Universität Dresden, D-01062 Dresden, Germany
* Correspondence: Muhannad.Al_Aiti@tu-dresden.de or Muhannad_Al_Aiti@outlook.de(M.A.A.); gheinrich@ipfdd.de (G.H.)

1. Molecular Characteristics of the Utilized Softwood Lignin

The composition of SBKL and the structural properties of Kraft Softwood lignin based on Southern Pine and Spruce extracted from the black liquor via the LignoBoost® concept are summarized in the Table S1.

Table S1. Structural properties of Softwood BioChoice™ Kraft Lignin and (*) Softwood Kraft Lignin obtained from the black liquor of Spruce + Pine wood and participated via the LignoBoost® concept [1] or a modified route thereof. **) Per 100-aromatic rings; ***) units in $mmol·g^{-1}$. (Number of digits after the decimal point are due to the references).

Reference	[2]	[3]*	[4]*	[5]*
**Composition**				
Klason lignin (wt. %)	91.1			96.0
Acid-soluble lignin (wt. %)	5.4		1.9	
Total lignin (wt. %)	96.5		97.5	
Ash (wt. %)	1.36		1.0	0.93
Total sugars (wt. %)	1.98		1.5	2.0
**Molecular Weight**				
Mw ($g·mol^{-1}$)	6772	5202	4470	6400
Mn ($g·mol^{-1}$)	949	1562	1000	1300
Mw/Mn (-)	7.1	3.3	4.5	5.0
**Functional Groups**				
Aliphatic -OH**	35	36.7	2.3***	
Phenolic -OH**	74	77.0	4.0***	
-OCH3	63			

-COOH	6	6.7	0.5***
α-Carbonyl	8	6.9	
**Elemental Analysis**			
C (%)	65.0	65.0	
H (%)	6.7	6.0	
O (%)	26.5	27.5	
N (%)	0.2	0.1	
S (%)	1.6	1.4	

2. Frequency Sweep Tests

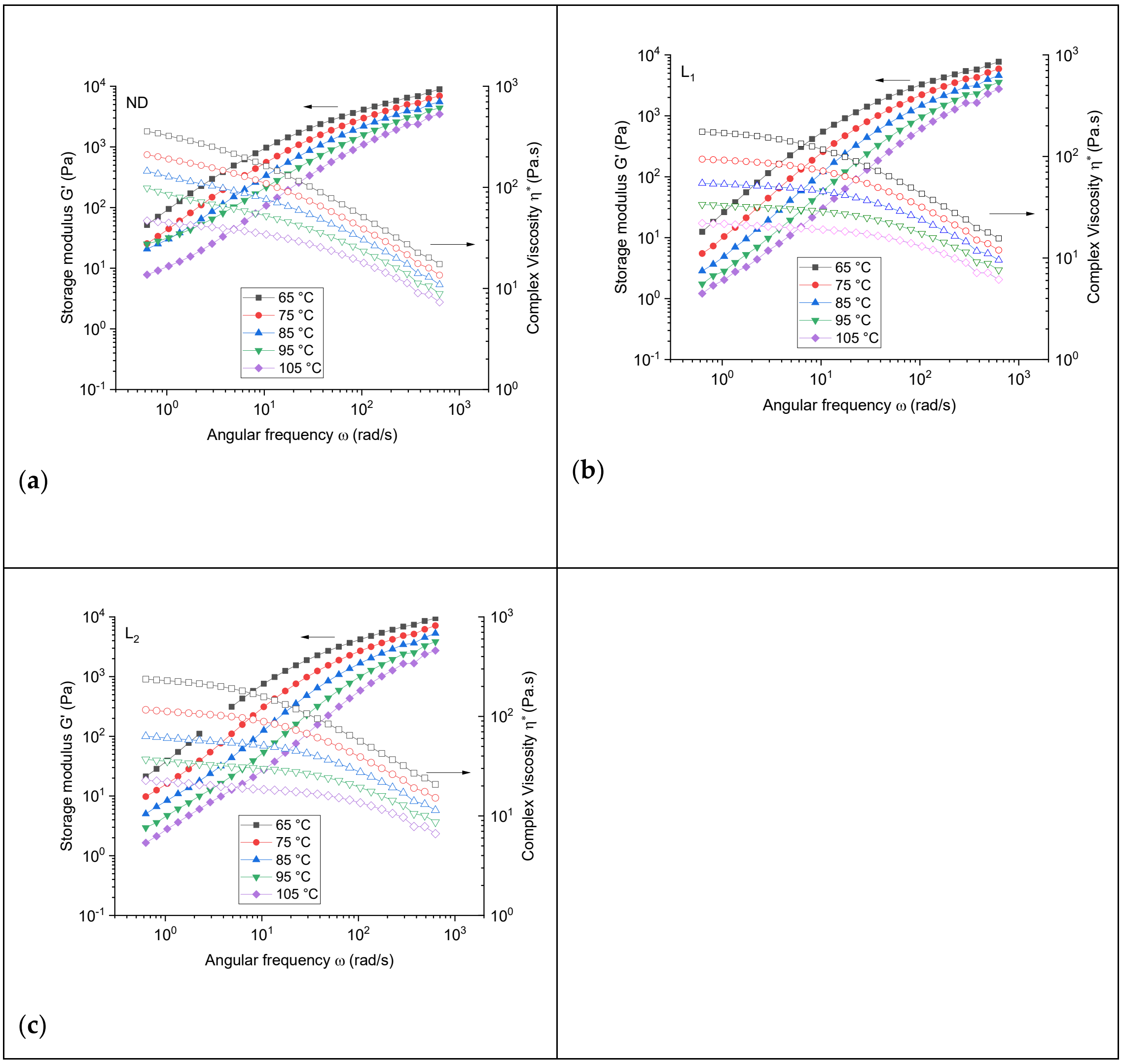

Figure S1. Frequency sweep tests of (**a**) dope ND; (**b**) dope L1; (**c**) dope L2 at different temperatures.

3. Spinnability

Prior to the spinnability measurements, the through-put of the spinning dopes was evaluated at different rotational speeds of the spinning pump. The lignin-containing dope showed comparatively lower through-put values at a lower rotational speed of the spinning pump (Figure S2a). At very low rotational speed, the mean through-put of the neat PAN dope (ND) and lignin containing dope (L1) was 0.117 and 0.09 $g \cdot min^{-1}$ at 85 °C, respectively. The spinnability was checked for both spinning dopes at different rotational (0.5; 1.0; 1.5; 2.0 rpm) speed set-ups of the spinning pump and different

take-up speed set-ups of the first take-up godets (2.5; 5.0; 10.0; 15.0; 20.0; 30.0 and 50.0 m·min-1. For the selected spinneret (L/D = 2; D = 300 µm) and at a spinning temperature of 85 °C and at a temperature of coagulation bath of 6 °C, the dope was considered as spinnable if continuous take-up was possible for 5 minutes without any breaks. Figure S2b and c illustrate the spinnability chart of both dopes ND and L1, respectively. Above a rotational speed limit of the spinning pump of 10 rpm, the system shows unstable and fluctuated through-put. Unfortunately, it was not possible to pursue the development of the dope pressure in the nozzle during the spinning. In contrast to the neat PAN dope (ND), lignin-containing dope (L1) showed many filament breakages (Figure S2c) at a low take-up speed. The fracture of the fibres occurred between the spinning nozzle and the first godet in a through-put and take-up speed dependent fashion as shown in Figure S3b,d.

At a take-up speed of 10 m·min^{-1} and rotational speed of the pump of 0.5 rpm, both dopes showed stable spinnability. The selected set-up was used to develop fibres with low diameter values (<25 µm) at high post drawing values (total DR = 10). Furthermore, the ability of the as-spun fibres to be stretched between the first and second godets was investigated considering the aforementioned set-up. The washing bath temperature between the first and second godet was set at 60 °C. Both dopes were in-line stretchable up to 4 times, after which, in the case of lignin containing fibres, the fibres got broken between the first and second godets. The in-line draw ratio was calculated from the term V2/V1. Where V1 is the take-up speed of the first godet and V2 is the take-up speed of the second godet.

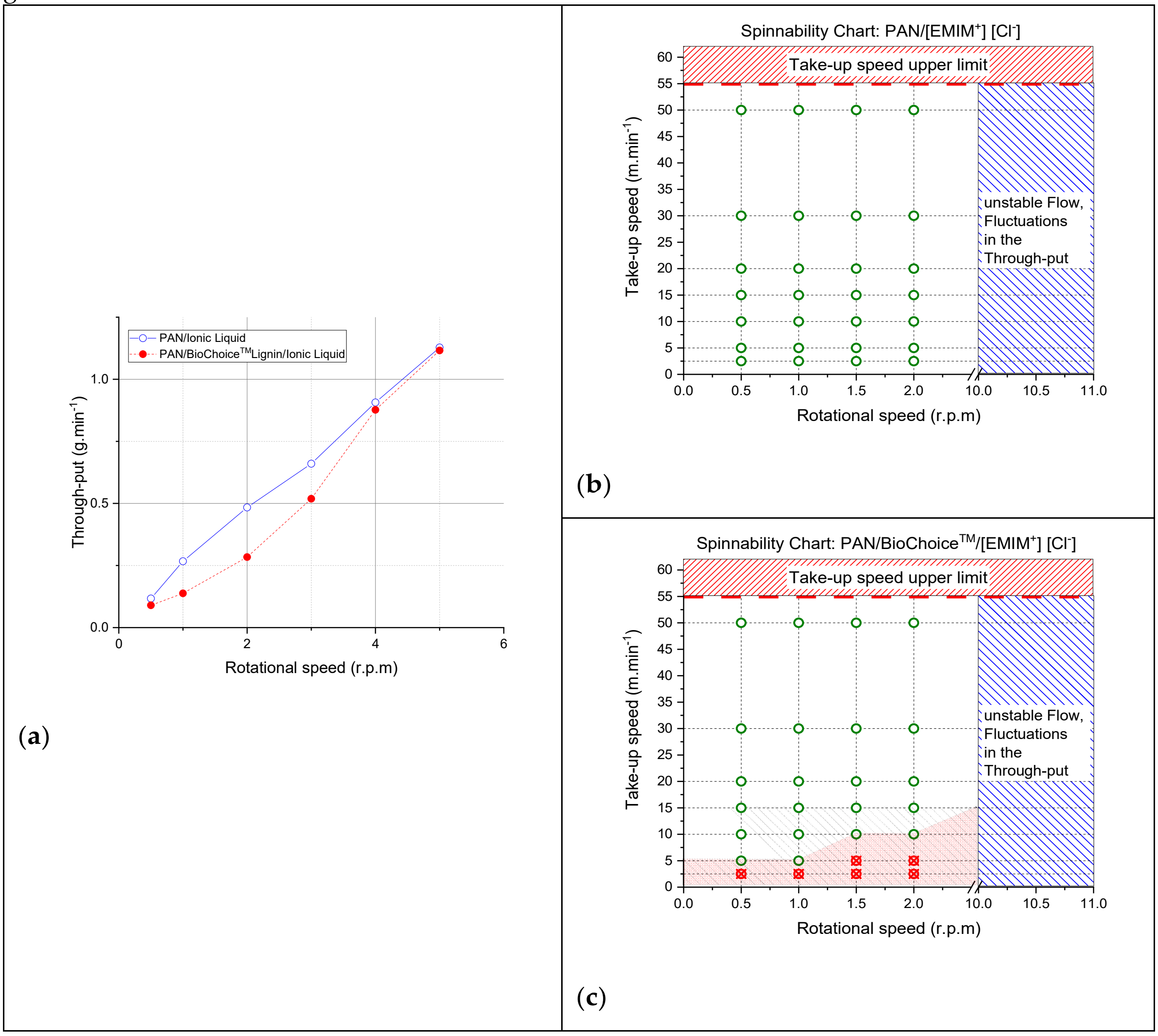

Figure S2. (**a**) Plot of the throughput of the up-scaled spinning dopes ND and L1 versus the rotational speed of the spinning pump; (**b**) the spinnability chart of the spinning dope ND and

(**c**) the spinnability chart of the spinning dope L1 (round green circles mean the system was found to be spinnable; and round tacked red circles for un-spinnable set-up.

4. Detailed DTG Curves of Unstabilized PAN and PAN/Lignin

Figure S3 illustrates in details the assignments of the Peaks ID 1, 2 and 3 for both PAN and PAN/Lignin based fibers in the DTG analysis under nitrogen for different heating rates.

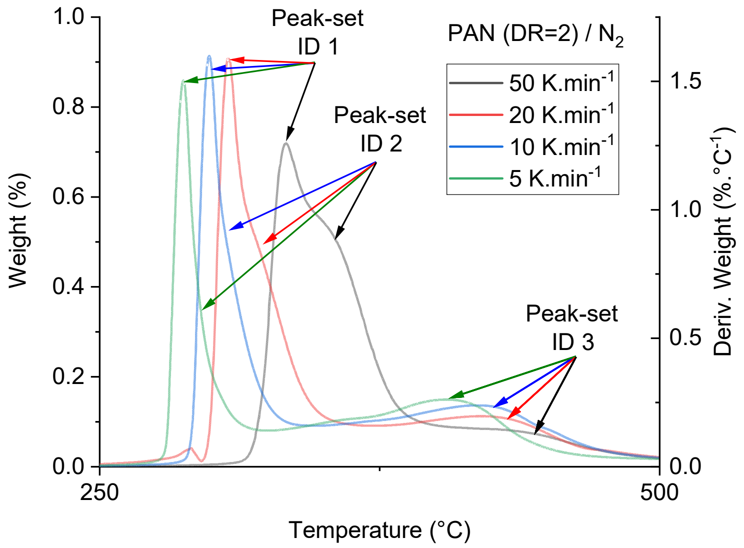

(**a**)

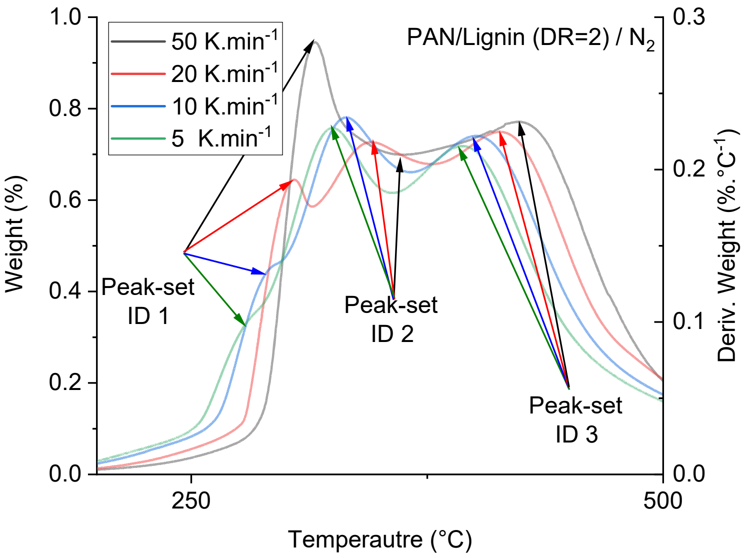

(**b**)

Figure S3. DTG curves of (**a**) PAN fibers and (**b**) PAN/lignin fibers (DR = 2) for detailed assignment of the Peak ID 1, 2 and 3 for both fiber types.

5. Scanning Electron Microscopy (SEM) and Energy-Dispersive X-Ray Spectroscopy (EDX)

Figure S4 illustrates in details high resolution SEM images of the fibres and high resolution EDX images of PAN and PAN/Lignin fibres at DR of 2.

**Type**	PAN Fibres (DR = 2)	PAN/Lignin Fibers (DR = 2)
**SEM**	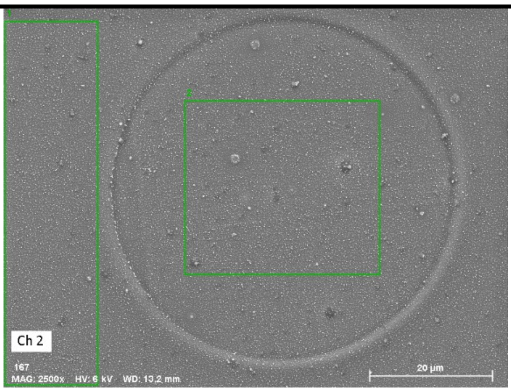 (**a**)	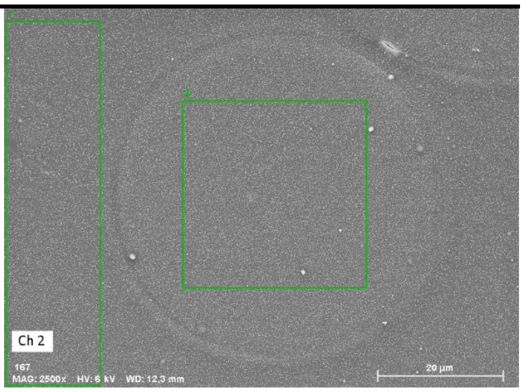 (**b**)
**EDX (Nitrogen)**	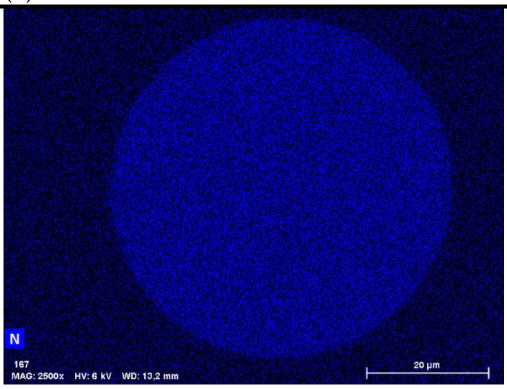 (**c**)	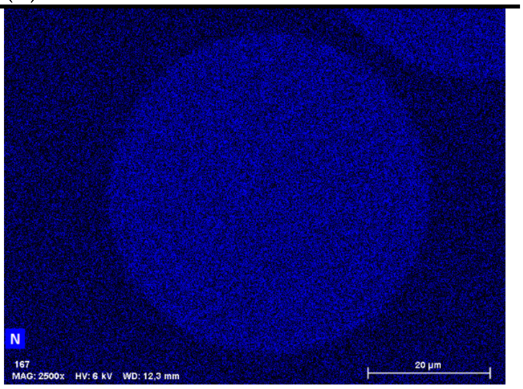 (**d**)
**EDX (Oxygen)**	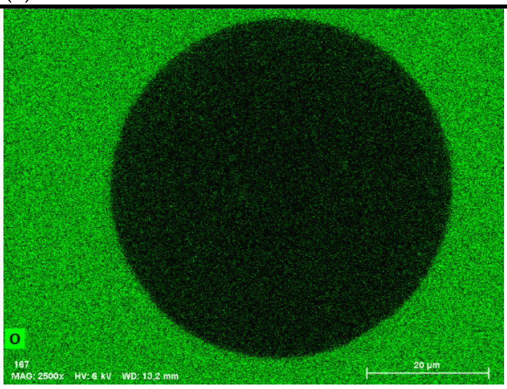 (**e**)	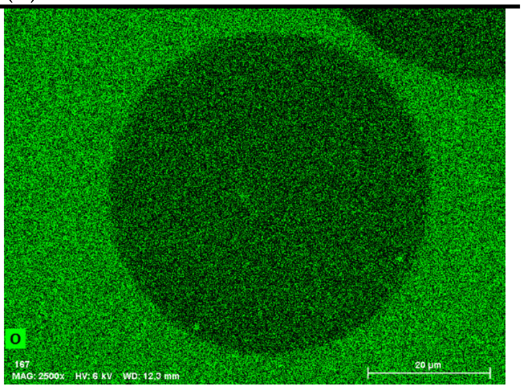 (**f**)
**EDX (Sulphur)**	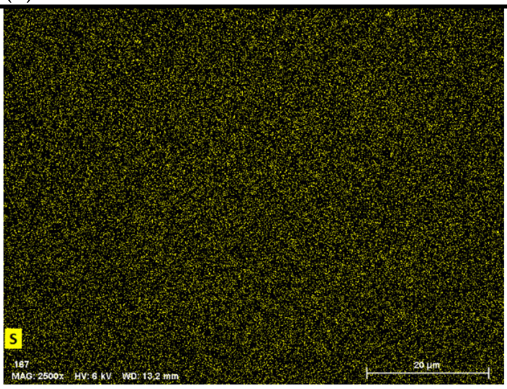 (**g**)	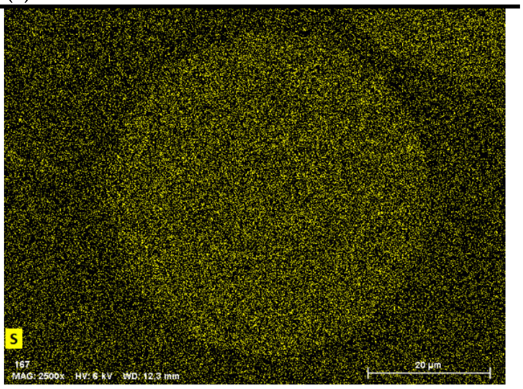 (**h**)

EDX (Chlorine)

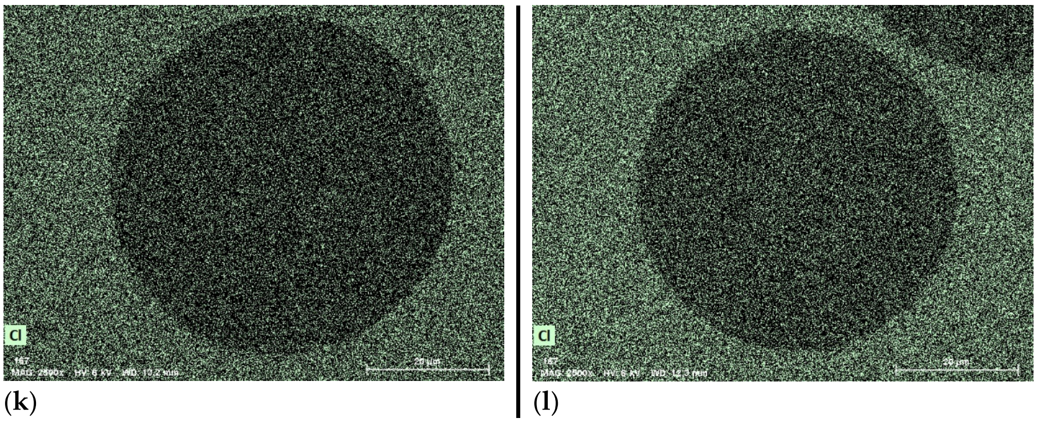

(**k**) (**l**)

Figure S4. SEM Images of (**a**) PAN and (**b**) PAN/Lignin fibres (DR = 2); Green rectangles indicate the areas selected for the EDX evaluations of the elemental concentrations of the atoms C, N, O, S and Cl. EDX images of nitrogen (**c**) and (**d**); oxygen (**e**) and (**f**); sulphur (**g**) and (**h**) and chlorine (**k**) and (**l**).

References

1. Tomani, P. *Cellul. Chem. Technol.* **2010**, *44*, 53.
2. Hu, Z.; Du, X.; Liu, J.; Chang, H.-m.; Jameel, H. *J. Wood Chem. Technol.* **2016**, *36*, 432–446.
3. Jiang, X.; Savithri, D.; Du, X.; Pawar, S.; Jameel, H.; Chang, H.-m.; Zhou, X. *ACS Sustain. Chem. Eng.* **2017**, *5*, 835–842.
4. Brodin, I.; Sjöholm, E.; Gellerstedt, G. *Journal* **2009**, *63*, 290.
5. Norberg, I.; Nordström, Y.; Drougge, R.; Gellerstedt, G.; Sjöholm, E. . *Appl. Polym. Sci.* **2013**, *128*, 3824–3830.

© 2020 by the authors. Licensee MDPI, Basel, Switzerland. This article is an open access article distributed under the terms and conditions of the Creative Commons Attribution (CC BY) license (http://creativecommons.org/licenses/by/4.0/).
